# Impact of postprandial glycaemia on health and prevention of disease

**DOI:** 10.1111/j.1467-789X.2012.01011.x

**Published:** 2012-10

**Authors:** E E Blaak, J-M Antoine, D Benton, I Björck, L Bozzetto, F Brouns, M Diamant, L Dye, T Hulshof, J J Holst, D J Lamport, M Laville, C L Lawton, A Meheust, A Nilson, S Normand, A A Rivellese, S Theis, S S Torekov, S Vinoy

**Affiliations:** 1 Department of Human Biology, School of Nutrition & Toxicology Research and Metabolism (NUTRIM), Maastricht UniversityMaastricht, the Netherlands; 2 Danone Research CenterPalaiseau, France; 3 Department of Psychology, University of SwanseaWales, UK; 4 Division of Applied Nutrition and Food Chemistry, Department of Food Technology, Engineering and Nutrition, Lund UniversityLund, Sweden; 5 Department of Clinical and Experimental Medicine, University Federico IINaples, Italy; 6 Diabetes Center, Department of Internal Medicine, VU University Medical CenterAmsterdam, the Netherlands; 7 Institute of Psychological Sciences, University of LeedsLeeds, UK; 8 Kellogg EuropeDen Bosch, the Netherlands; 9 Department of Biomedical Sciences and Novo Nordisk Foundation Centre of Basic Metabolic Research, University of CopenhagenCopenhagen, Denmark; 10 Centre de Recherche en Nutrition Humaine, Rhône-Alpes, Center for European Nutrition, Safety and Health, Centre Hospitalier Lyon SudLyon, France; 11 ILSI EuropeBrussels, Belgium; 12 Südzucker/BENEO GroupObrigheim, Germany; 13 Kraft Foods, R&D Centre, Nutrition DepartmentSaclay, France

**Keywords:** Health, physiological effects, postprandial glycaemia, risk factors

## Abstract

Postprandial glucose, together with related hyperinsulinemia and lipidaemia, has been implicated in the development of chronic metabolic diseases like obesity, type 2 diabetes mellitus (T2DM) and cardiovascular disease (CVD). In this review, available evidence is discussed on postprandial glucose in relation to body weight control, the development of oxidative stress, T2DM, and CVD and in maintaining optimal exercise and cognitive performance. There is mechanistic evidence linking postprandial glycaemia or glycaemic variability to the development of these conditions or in the impairment in cognitive and exercise performance. Nevertheless, postprandial glycaemia is interrelated with many other (risk) factors as well as to fasting glucose. In many studies, meal-related glycaemic response is not sufficiently characterized, or the methodology with respect to the description of food or meal composition, or the duration of the measurement of postprandial glycaemia is limited. It is evident that more randomized controlled dietary intervention trials using effective low vs. high glucose response diets are necessary in order to draw more definite conclusions on the role of postprandial glycaemia in relation to health and disease. Also of importance is the evaluation of the potential role of the time course of postprandial glycaemia.

## Introduction and background

Carbohydrates are, on a weight basis at least, the most important nutrient of the human diet, after water. In terms of fulfilling energy requirements, they should also be the major component of the diet according to nutritional guidelines: carbohydrates should represent 45–55% of our daily energy intake (EI), 10–15% being simple carbohydrates or sugars, the remainder being starches and oligosaccharides. Carbohydrates present a daily metabolic challenge for the body, as glucose is the prime fuel normally used by brain cells and many organs. Although the brain and organs rely on glucose for their energetic needs, the body can only store, mainly in the liver and muscles, up to about 1 kg of glucose, which represents no more than 2 d of consumption. To manage this metabolic challenge, there is complex machinery balancing the influx of carbohydrates from the diet digested in the gut to the cells, partly for storage and partly for immediate oxidation. Blood glycaemia reflects this balance, and there are two hormonal systems to limit, on the one hand, the increase of blood glycaemia and, on the other, the decrease to ensure a proper permanent delivery of glucose to cells.

Postprandial glycaemia (and related insulinaemia and lipidaemia) has been implicated in the aetiology of chronic metabolic diseases such as type 2 diabetes mellitus (T2DM) and cardiovascular disease (CVD). Obesity is the most important risk factor for the development of these metabolic diseases. The worldwide prevalence of overweight (body mass index [BMI] ≥ 25 kg m^−2^) and obesity (BMI ≥ 30 kg m^−2^) has risen dramatically in recent decades and is still on the increase. Raised BMI is a major risk factor for chronic diseases such as CVD (mainly heart disease and stroke), type 2 diabetes (also rapidly becoming a global epidemic), musculoskeletal disorders (especially osteoarthritis) and some cancers (e.g. endometrial, breast and colon) (1,2). Obesity and related health complications lead to reduced quality of life, massive healthcare costs and ultimately premature death, indicating the urgency for the identification of simple cost-effective strategies for prevention and management.

Pharmacological manipulation of postprandial glucose by acarbose, an alpha-glucosidase inhibitor, may decrease progression to diabetes by 25% (3). However, manipulation of postprandial glucose by nateglinide – which stimulates insulin release – was unable to reduce diabetes incidence (NAVIGATOR trial [4]), suggesting that the mechanisms of action for reducing postprandial glucose may be a determinant of the final effect. Furthermore, recent large-scale lifestyle intervention studies indicate that lifestyle intervention in impaired glucose-tolerant subjects improves glucose tolerance and reduces diabetes risk by 30–60% (5–7). The underlying mechanisms relating reduced postprandial glucose to the prevention of disease are less clear. The level of postprandial glycaemia has been implicated in body weight control either via an effect on appetite or via an effect on nutrient partitioning of markedly increased insulin and glucose concentrations (8). The latter may promote fat storage in adipose and non-adipose tissues through effects on adipose tissue lipolysis and skeletal muscle fat oxidation. Greater postprandial fat storage in the liver and skeletal muscles has been associated with the development of insulin resistance (IR) (9). There are increasing indications that both upward and downward fluctuations of post-meal glucose concentrations may be particularly important in the aetiology of chronic disease. It has been hypothesized that a chronic high early glycaemic response followed by late depression of blood glucose below baseline levels (reactive hypoglycaemia) and increased free fatty acid (FFA) concentrations is associated with the development of IR (10). Addtionally, fasting and postprandial glucose concentrations represent a strong risk factor for the development of T2DM and CVD by promoting oxidative stress, inflammation and endothelial dysfunction (as reviewed in the sections on oxidative stress and inflammation, on the prevention of diabetes and IR and on cardiovascular prevention in this article).

**Figure 1:**
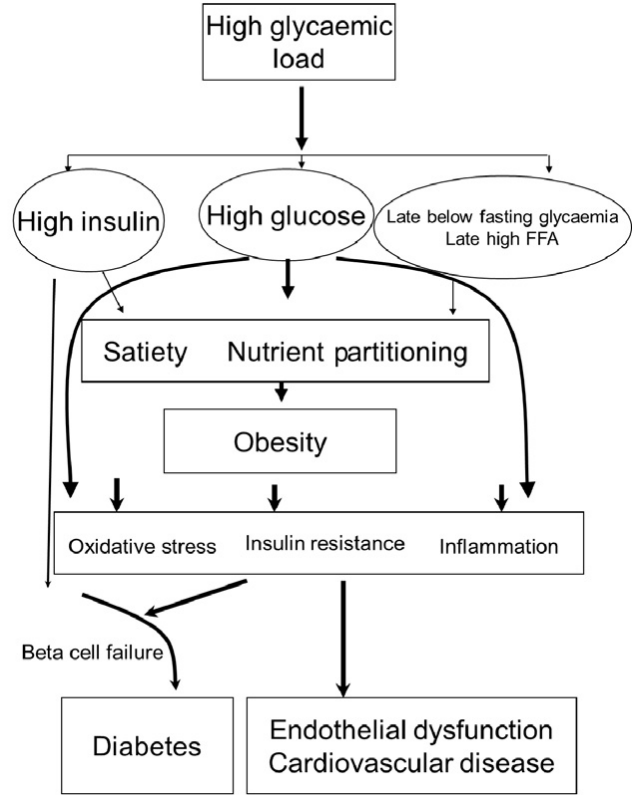
Putative relationships between postprandial glycaemia and risk factors for obesity, diabetes and cardiovascular disease. FFA, free fatty acid.

The scope of the present overview is, firstly, to discuss the available evidence on the role of postprandial glucose in relation to body weight control, the development of oxidative stress, diabetes and CVD. Figure 1 gives an overview of the putative relationships between postprandial glycaemia and chronic disease, which will be addressed in the different sections of this review. Subsequently, physical exercise is addressed as an important strategy to manage postprandial blood glucose within ‘more healthy levels’. Finally, chronic diseases such as obesity and diabetes are increasingly being associated with cognitive decline. Glucose forms the major energy substrate of the brain and is central to the normal functioning of the central nervous system. The available knowledge on postprandial glucose response in relation to cognitive functions is, therefore, addressed in the final section of this article, which ends with a brief overall discussion.

As carbohydrate is the main dietary component affecting glycaemia, many studies that are described involve the effects of dietary carbohydrate. Both the amount and type of carbohydrate have an effect on both postprandial glycaemia and insulinaemia, with differences not explained by glucose chain length. In 1981, the concept of glycaemic index (GI) was introduced by Jenkins *et al*. (11) to quantify the glycaemic response to carbohydrates in different foods. Glycaemic load, the mathematical product of the GI of a food and its carbohydrate content, has been proposed as a global indicator of the glucose response and insulin demand induced by a serving of food. In practice, these reflect a total glycaemic response to a food or diet regardless of the food components responsible or the shape of the glycaemic response. In addition to these factors, the shape of the glycaemic curve is studied using different measures involving the total glucose response, the incremental response, the maximum amplitude of glucose excursions, the mean amplitude of glycaemic excursions (MAGE) index or the standard deviation (SD) around the mean glucose value. The number of different measures of glycaemic variability illustrates that it is a complex phenomenon including both intraday and interday variability. Finally, the question as to whether the relationship between glycaemia, health and disease involves a continuous relationship, or if there are clear thresholds for effects, will be specifically addressed because this issue is important for disease prevention.

## Postprandial glycaemia and weight management^1^

### Introduction

The aim of this section is to collate and evaluate the evidence for the impact of postprandial glycaemia on weight management in healthy (non-diabetic) subjects. The evidence for the effects of postprandial glycaemia on obesity prevention comes from both short (≤1 d) and longer-term studies (≥8 d) investigating the effects of dietary interventions on appetite control and body weight. Additionally, there are indications that postprandial glycaemia may affect body weight control by affecting the balance between fat and carbohydrate oxidation, thereby affecting energy expenditure, but this mechanism is probably of lesser importance than appetite control in the regulation of body weight. The capacity of any food to suppress hunger and inhibit eating is mediated by processes that can be roughly classified as sensory, cognitive, post-ingestive (but pre-absorptive) and post-absorptive. Together these processes have been referred to as the satiety cascade (12,13). The satiety cascade embodies the important distinction between satiation and satiety, the two main mechanisms upon which appetite control is based. Satiation can be defined as the process or set of processes that bring a period of eating to a close (or intra-meal satiety). Satiety, however, is typically defined as the state of inhibition over further food intake once a period of eating has ended (or inter-meal satiety). Satiety is therefore a short-term parameter that is generally not assessed over the long-term. Although enhanced satiety might improve tolerance to or compliance wth consumption of energy-reduced foods, a change in hunger or fullness (for example) will only be of clinical significance for weight control if it can be translated into a decrease in actual EI that results in either weight loss or maintenance over time. Short-term studies have the disadvantage of not predicting food intake or benefits related to increased dietary tolerance or compliance during the next days or weeks if the diet is continued. It is, therefore, necessary to examine the long-term effects of dietary manipulations on body weight to determine if short-term effects of certain foods/food components on satiety translate into weight loss. Blundell and colleagues (14) have now provided guidance on good practice when carrying out research to assess the impact of foods and food ingredients on the expression of appetite. This is especially important in the light of the regulatory procedures to be implemented regarding satiety and appetite claims on foods.

### Postprandial glycaemia and appetite control: evidence from short-term (acute) dietary intervention studies

#### Short-term effects of GI interventions on postprandial glycaemia and satiety

It has been suggested that low GI (LGI) foods increase satiety compared with high GI (HGI) foods (15,16). Many short-term studies (lasting for a single meal or a single day) have addressed the question of whether consumption of LGI foods reduces hunger and/or promotes satiety relative to consumption of HGI foods. Table 1 summarizes the results of these acute studies on appetite control in healthy participants.

**Table 1:** Effects of short-term human intervention studies examining the effect of GI interventions on appetite control in healthy participants

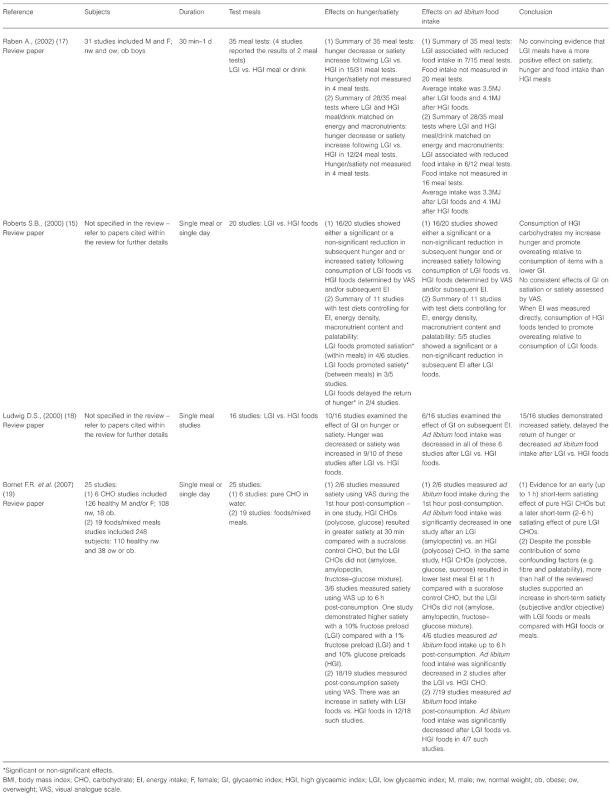

Raben (17) conducted a systematic review of published human intervention studies comparing the effects of HGI and LGI foods or diets on appetite, food intake, energy expenditure and body weight (see Table 1 for details of the short-term studies reviewed). Within this she reviewed the results of 31 short-term studies (<1 d) and 20 longer-term studies (<6 months; see section on long-term effects of GI interventions on postprandial glycaemia and body weight control). The short-term studies comprised a total of 35 different meal tests (because 4 studies reported the results of 2 separate meal tests). Overall, Raben concluded that the data from the short-term human intervention studies included in her review did not provide convincing evidence that LGI meals have a more positive effect on satiety, hunger and food intake than HGI meals. Around this time a couple of other reviews (15,18) were more positive in their conclusions regarding LGI foods and appetite, but these included fewer studies (16 and 20 meal tests, respectively).

Bornet *et al*. (19) conducted a systematic review that examined 32 human studies that had investigated the relationship between the glycaemic response to foods and its impact on satiety and weight management (Table 1). Both the 25 short-term studies (1 d or less) with liquid carbohydrates (e.g. glucose or fructose in water) or foods and longer-term studies (2 weeks–6 months; see section on long-term effects of GI interventions on postrandial glycaemia and body weight control) were included.

Bornet and colleagues (19) concluded that whatever the method used to assess satiety (subjective or objective) and despite the possible contribution of some confounding factors (e.g. fibre and palatability), more than half of the reviewed studies supported an increased short-term satiety with LGI foods or meals compared with HGI foods or meals. In addition, this result was not contradicted by those studies that were inconclusive.

Limited research in humans suggests that slowly digested starch may blunt the postprandial increase and subsequent decline of blood insulin and glucose concentrations, leading to prolonged satiety, compared with more rapidly digested starch. A study by Sands and colleagues (20) examined the postprandial metabolic and appetite responses to 50 g of available carbohydrate given as waxy maize starch (WM, a slowly digested starch), a maltodextrin-sucrose mixture (MS, rapidly digested carbohydrate) or white bread (control). Subjects were 12 (6 men, 6 women) young, healthy, normal-weight, insulin-sensitive adults. Postprandial plasma glucose and insulin, energy expenditure, and subjective appetite (hunger, fullness and desire to eat using 13-point linear scales) were measured over 4 h. The authors found that consumption of the slowly digested WM starch, compared with MS, produced blunted 4-h plasma glucose and insulin responses (*P* < 0.001). However, postprandial energy expenditure and appetite responses did not differ between the WM and MS treatments. These results are similar to those of Wachters-Hagedoorn *et al*. (21), who reported that 50 g of available carbohydrate from uncooked corn starch (a slowly digested starch) led to smaller glucose and insulin area under the curve (AUC) compared with 50 g of glucose. Slowly digested starches may therefore be a suitable choice of carbohydrate when a slower, more prolonged period of glucose absorption is desired, and may eliminate the potential for depression of blood glucose below baseline levels (reactive hypoglycaemia) after the ingestion of a quickly digested food. Although the study by Sands and colleagues (20) did not find differences in hunger or satiety among the study treatments, the authors acknowledge that their results should be viewed with caution until confirmed (because of the possible confounding effect of food form/palatability). Granfeldt *et al*. (22), for example, studied the satiety responses to slowly digested barley products compared with a white bread control. The barley products were found to elicit greater satiety than white bread, and this was found to be inversely correlated with lower glucose responses. Additionally, recent results with cereal-based breakfasts have shown that the magnitude of reactive hypoglycaemia (i.e. depression of blood glucose below baseline levels) was negatively correlated to subjective rating of satiety in the late postprandial phase. Also, a low incremental insulin peak after breakfast was associated with less severe reactive hypoglycaemia and with a milder recovery of plasma ghrelin (which rises with hunger) (23). In a follow-up study highlighting rye breakfast meals, a higher glycaemic profile (glycaemic peak, defined as the quota between glycaemic peak concentration and time duration of glycaemia above fasting level, and measured as min mmol^−1^ L^−1^), was related to a lowered *desire to eat* before lunch (AUC 210–270) and to a lower concentration of ghrelin in the late postprandial phase (270 min), which in turn related to a lower voluntary EI at a subsequent buffet lunch meal (24).

#### The influence of postprandial glycaemic responses *per se* on satiety in the short-term

Although it has been suggested that foods with an LGI increase satiety (25,26), the evidence that glycaemic responses *per se* influence satiety is not clear. Wolever *et al*. (27) state that neither the timing nor the direction of the effect of LGI foods on satiety or food intake is consistent and that results are often confounded by lack of control of variables other than glycaemic response, which could account for increased satiety, such as dietary fibre (26), protein (28) and meal volume (29). The glucose-lowering effects of dietary fibre, protein and fat are discussed in the section ‘Effects of other dietary factors on postprandial glycaemia in both the short- and the long-term’.

Wolever and colleagues (30,31) found that the glycaemic responses elicited by exactly the same test meal vary from day to day within subjects with a mean coefficient of variation of ∼25% in normal subjects. In view of this, Wolever and colleagues (27) postulated that if glycaemic responses affect satiety then variations in response after the same test meal should be associated with changes in satiety. They designed and carried out a study to test this hypothesis but found that the day-to-day variation in glycaemic response elicited by white bread was not related to variation in satiety in non-obese subjects. The researchers concluded that the effect of LGI foods on short-term satiety and food intake is due to factors other than blood glucose response (e.g. fibre and protein content). This conclusion is also shared by Peters and co-workers (32), who examined the postprandial glucose, insulin and appetite responses to drinks differing only in rate and digestibility of carbohydrate. This was achieved by comparing different glucose polymers: maltodextrin (MDX, rapidly digestible), medium-chain pullulan (MCP, slowly but completely digestible) and long-chain pullulan (MLP, indigestible). It was hypothesized that the MCP would result in a more sustained postprandial blood glucose and plasma insulin response, leading to a reduction in appetite as compared with MDX. However, although the digestibility, glucose and insulin data confirmed the rapid, slow and indigestible nature of MDX, MCP and MLP, respectively, only MLP reduced appetite compared with the MDX control, whereas the MCP did not. Hence, glycaemic responses *per se* were found to have minimal effects on appetite when tested in products differing only in the rate and digestibility of carbohydrate.

In an attempt to clarify the roles of blood glucose and insulin in short-term appetite regulation, Flint and colleagues (33) conducted a meta-analysis of all test meal studies having individual participant data (IPD) on blood glucose, insulin and subjective appetite sensations (using visual analogue scale) conducted in their department (between 1990 and 2003). Seven single test meal studies with a total of 136 (131 males, 5 females) healthy normal-weight (*N* = 92) or overweight participants (*N* = 44, all male) were included. All test meals were served as breakfast after an overnight fast. In six of the seven studies, the participants were served an *ad libitum* lunch meal at the end of the postprandial period (180–315 min). In each study, insulin, blood glucose and subjective appetite (hunger/satiety) were measured frequently during the postprandial period.

Data were analysed by fixed-effects study level (SL) meta-regression analysis and IPD regression analysis on all subjects and as subgroup analyses on normal-weight and overweight participants separately. In the SL analysis a greater postprandial insulin response was associated with decreased hunger in all subjects and in the normal-weight and overweight subgroups (*P* < 0.019). Furthermore, a greater postprandial insulin response was associated with increased satiety in normal-weight subjects (*P* = 0.004) and with lower EI from the *ad libitum* lunch (EI) in overweight subjects (*P* = 0.022). Multivariate IPD analysis showed similar associations but only in the normal-weight subgroup for hunger, satiety and EI (*P* < 0.028) and in all subjects for EI (*P* = 0.016). The only association involving blood glucose was the multivariate IPD analysis showing a negative association between blood glucose and EI in all subjects. These results suggest that insulin, but not glucose, is associated with short-term appetite regulation in healthy subjects but that the relationship is disrupted in overweight and obese subjects. The authors concluded that the postprandial insulin response may be an important satiety signal and that central nervous system IR in overweight might explain the blunted effects on appetite. Hence, the results of this study do not support a significant role for blood glucose in short-term appetite regulation, a finding supported by other studies (34–36). However, it has been proposed that the potential effect of insulin as a satiety signal might require raised blood glucose concentrations (37). Hence, the presence of both hyperinsulinaemia and hyperglycaemia (or an interaction between the two) may be required to inhibit appetite and food intake. Further studies are also required to clarify how insulin and the incretin hormones (glucose-dependant insulinotropic polypeptide [GIP] and glucagon-like peptide-1 [GLP-1]) interact in the regulation of appetite.

### Postprandial glycaemia and body weight control: evidence from long-term dietary intervention studies

#### Long-term effects of GI interventions on postprandial glycaemia and body weight control

Table 2 summarizes the findings from the available long-term studies of the effects of GI interventions on body weight control in healthy participants. Raben (17) reviewed 20 longer-term studies (<6 months duration) of the effects of LGI or HGI on body weight (see Table 2 for study details). Eighteen of these studies used weight maintenance (isoenergetic) or weight-loss (energy restricted) diets, and the remaining 2 longer-term studies used *ad libitum* diets. Taken together, the results of the 20 longer-term studies reviewed by Raben revealed a greater weight loss on an LGI diet in 4 studies, a greater weight loss on an HGI diet in 2 studies and no differences in weight loss between these diets in 14 studies. The mean weight change across all 20 studies was −1.5 kg on an LGI diet and −1.6 kg on an HGI diet. When focusing only on the nine studies where energy and macronutrient content were similar, one study found a decrease in body weight on an LGI diet vs. an HGI diet, but the other eight studies did not. Raben (38) concluded that LGI diets were not superior to HGI diets with respect to long-term body weight control but that the optimal study with *ad libitum* EI on diets matched in all aspects except GI (e.g. macronutrients, fibre, energy density, food form) is still to be performed.

**Table 2:** Long-term human intervention studies examining the effect of GI interventions on body weight control in healthy participants

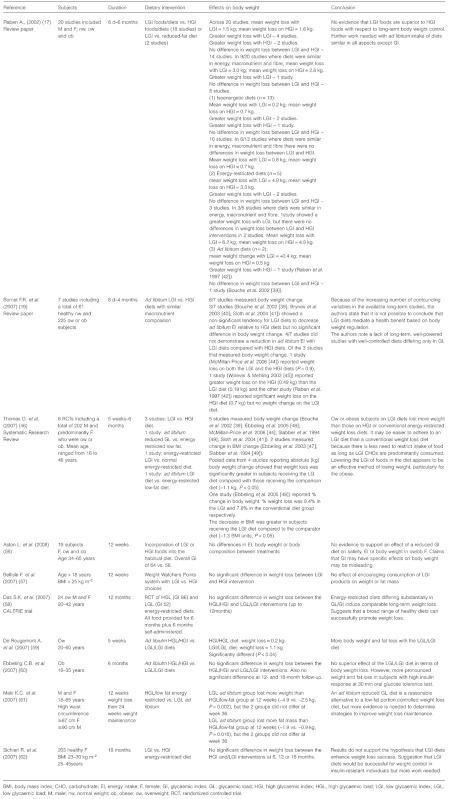

Bornet and colleagues (19) argue that only *ad libitum* diet design studies are relevant for evaluating the effect of LGI vs. HGI diets on spontaneous long-term regulation of EI and body weight. Consequently in their review, the only long-term studies that were selected for analysis were those in which LGI and HGI diets were consumed *ad libitum*. In addition, only studies for which the proportion of macronutrients was similar between the LGI and HGI diets were retained for final analysis in order to take into account the known different satiating capacities between macronutrients. Bornet and colleagues identified seven longer-term studies meeting the above selection criteria (two of which were included in Raben's review; see Table 2 for study details). Three of these studies (39–41) demonstrated a non-significant tendency of LGI diets to induce a decrease in *ad libitum* EI compared with HGI diets with no significant difference in body weight change. In the four remaining *ad libitum* diet studies identified by Bornet and colleagues (19), the LGI diets did not reduce EI relative to the HGI diets (42–45). Bornet and colleagues concluded that while there was some evidence from short-term studies that low-glycaemic foods (LGI in particular) possessed greater satiating properties than high-glycaemic foods, the available long-term studies did not allow a conclusion about the regulation of body weight and that more long-term, well-powered studies with well-controlled diets differing only in GI were needed.

Thomas and colleagues (46) conducted a systematic review of the effects of LGI or low glycaemic load (LGL) diets on weight loss in healthy overweight or obese people. Randomized controlled trials (RCTs) comparing an LGI or an LGL diet with a higher GI or GL diet or other control diet (Cdiet) in overweight or obese participants were selected for inclusion. The authors identified six RCTs meeting their inclusion criteria with interventions varying from 5 weeks to 6 months duration with up to 6 months follow-up after the intervention period. Three of the studies identified by Thomas and colleagues were also included in the review by Bornet and colleagues (39,41,44). Thomas and colleagues also identified additional studies by Ebbeling and co-workers (47,48) and Slabber and co-workers (49).

Thomas and colleagues (46) found that the decrease in body mass, total fat mass and BMI, total cholesterol and low-density lipoprotein (LDL)-cholesterol was significantly greater in healthy overweight and obese subjects receiving the LGI compared with Cdiets (including HGI or high glycaemic load [HGL] diets and conventional energy-restricted, low-fat weight-loss diets) even when the diets were *ad libitum*. Of the six studies included in this review, two studies included obese participants (47,49) and compared LGI or LGL diets with conventional weight-reducing low-fat diets. The remaining four studies included participants with borderline normal weight (BMI = 25) or overweight (BMI of 26–30) and compared an LGI or an LGL diet with a higher GI or GL diet. Only one study involved children (47). In the two studies where all the subjects were obese, the effects of the LGI or LGL diets were more apparent. Hence, the authors concluded that lowering the GI of foods in the diet appears to be an effective method of losing weight, particularly for the obese. However, Thomas and colleagues (46) do acknowledge that the major challenge in weight management is the sustainability of weight loss or the maintenance of body weight. Consequently they concede that longer trials with increased lengths of follow-up (>6 months) are needed to determine if the improvements reported with LGI diets can be maintained and incorporated into lifestyle long-term.

Hare-Bruun *et al*. (50) have examined the epidemiological evidence linking GI and GL to heart disease, insulin sensitivity (T2DM) and obesity among initially healthy people. The observational studies of associations between GI and BMI, body weight or body composition were primarily cross-sectional and conducted among adults. The authors found that the evidence for associations between GI, and particularly GL, and health (taking into account the magnitude of obesity) among free-living populations is mixed. These results contrast with those of Thomas *et al*. (46) and also Livesey *et al*. (51,52), who observed reductions in body weight on both LGI and LGL diets. However, a major difference between these two reviews and the review by Hare-Bruun and colleagues was the focus on treatment studies and hence weight loss among overweight and obese people in the meta-analyses. Furthermore, the review by Livesey and colleagues included studies in subjects with type 1 diabtes mellitus (T1DM) or T2DM, impaired glucose tolerance (IGT), hyperlipidaemia and coronary heart disease (CHD) risk. Instead, the studies reviewed by Hare-Bruun and colleagues focused on the prevention of weight gain and reported on the associations between the GI and GL of the habitual diet and weight change over longer time periods.

As the evidence from their review was not strong, Hare-Bruun *et al*. (50) concluded that it seems premature to include GI in the dietary recommendations for healthy populations. They also argue that it may be difficult for individuals to apply the GI concept to the usual diet as this requires some nutritional knowledge or at least a dedicated effort to learn how to use it in practice. In response to this type of concern, Pawlak and colleagues (53) have emphasized that several studies have reported significant improvement in relevant end points among subjects consuming self-selected LGI diets. Moreover, LGI diets not only facilitated glycaemic control but were also perceived as ‘simple and practical’ by adults and children with newly diagnosed diabetes (54,55).

The meta-analysis published by Livesey and colleagues (51,52) included controlled GI intervention studies published until 2005. Since then, several additional RCTs on GI/GL in healthy overweight or obese subjects have been published (56–62). The difference in weight loss observed between the high- or LGI/GL diets across these studies ranged from 0.5 kg (57) to −1.9 kg (61) and was statistically significant in only one study (59). In a review on sugar consumption and body weight, which included these new studies, Van Baak & Astrup (63) conclude that any effect of LGI/GL diets on body weight appears to be small and is, therefore, of limited practical and clinical importance.

More recently, data from the large-scale intervention study on Diet, Obesity and Genes (Diogenes) (64–66) (http://www.diogenes-eu.org) have become available. In the Diogenes study, overweight adults from eight European countries who had lost at least 8% of their body weight on an 800-kcal low-calorie diet (LCD) were randomized to one of five *ad libitum* diets to prevent weight regain over a 26-week period. Maintenance diets were either high or low in protein and GI according to a two-by-two factorial design or a Cdiet (following the dietary guidelines in each participating country). The recently published results demonstrate that both the rate of completion of the dietary intervention phase and the maintenance of weight loss were highest in participants assigned to the high-protein (HP) diets and to the LGI diets. In addition, participants assigned to the HP/LGI diet continued to lose weight after the LCD weight loss phase (65). Hence, in this study, a modest increase in protein content and a modest reduction in GI led to an improvement in study completion and maintenance of weight loss, suggesting that an HP/LGI diet is best for weight maintenance after weight loss.

#### Effects of other dietary factors on postprandial glycaemia in both the short- and the long-term

Although many studies have been performed with variable results, many are confounded by dietary differences in the diets other than glycaemic response alone (energy, macronutrient and fibre content) and as such are difficult to interpret. Three well-controlled studies matching both macronutrients and fibre (Bouche *et al*. [39] 5 weeks; Sloth *et al*. [41] 10 weeks; Sichieri *et al*. [62] 18 months) found no change in body weight or body fat mass. In addition, Aston *et al*. (56) performed an RCT in overweight and obese women, measuring the effect of a higher vs. a lower GI *ad libitum* diet on appetite, dietary intake and body weight using a cross-over design (12 weeks on each diet). The diets were matched for energy, macronutrient and fibre intakes, but no differences were found between the higher and lower GI diets for any of the end points measured. These findings indicate that the difference in appetite and body weight sometimes observed after the consumption of LGI foods relative to HGI foods is due to factors other than the difference in blood glucose (27,32). Based on her review of the relevant literature, Raben (17) argues that postprandial glycaemia and appetite are not related. Peters and colleagues suggest that inconsistencies between studies examining the effects of GI on appetite might be explained by a lower-than-expected carbohydrate energy availability or by other (physical and nutritional) food properties differing between test products (43).

In addition to the quantity and digestibility of starches and the quantity and sources of sugars in a meal, many other factors influence the glycaemic response to foods. These include the presence of fat and protein, the degree of processing and cooking, and the amount and types of dietary fibre present. Indeed, it is often not possible to distinguish the effects of lowering postprandial glycaemia *per se* from the known effects of certain dietary fibres on appetite and body weight (67,68). A number of mechanisms have been suggested to account for the beneficial effects of certain fibres on appetite and weight control. These mechanisms could be due to an action in the mouth, stomach or intestines. Specific soluble fibres in particular could increase the viscosity of the diet and slow down digestion, thus stimulating the release of satiety peptides. Some fibres could also act as a mechanical barrier to the digestion of other macronutrients, leading to blunted glucose and insulin responses. Additionally, fermentation products of certain fibres in the large intestine, including specific short-chain fatty acids (SCFAs), may exert a late action on satiety.

It is generally accepted that adding fat and/or protein to carbohydrate reduces blood glucose levels compared with carbohydrate alone (69). Co-ingestion of fat with carbohydrate slows gastric emptying and thus the release of glucose into the blood, ultimately lowering the GI. Co-ingestion of protein with carbohydrate is also known to reduce postprandial glycaemia (70–72). Milk proteins, for example, reduce blood glucose responses when consumed with carbohydrates (72) and are consistently found to be insulinaemic. The exact mechanisms associated with the glucose-lowering effects of fat and protein are not clear, although they are suggested to occur through similar mechanisms such as delayed gastric emptying (73) and/or enhanced insulin secretion through augmented GIP and GLP-1 secretion (74). However, fat and protein might act to modulate the postprandial glucose response via different mechanisms. Hence, in one human study protein had a greater (two- to three-fold) hypoglycaemic action than fat (75).

### Postprandial glycaemia and substrate oxidation

It has been hypothesized that LGI foods may affect body weight control and insulin sensitivity by promoting satiety and stimulating fat oxidation at the expense of carbohydrate oxidation (8). Indeed, animal studies show that a reduced GI can shift postprandial substrate use in favour of fat oxidation, independent of diet-induced changes in body composition or EI (76–78). It was recently shown that a reduced glycaemic response after a mixed meal containing isomaltulose (ISO) instead of sucrose (SUC) may improve fat oxidation rates at the expense of carbohydrate oxidation in overweight subjects (79). Similar findings were also observed during exercise conditions (80). As indicated above, this shift towards fat oxidation may be of practical use for body weight control. A lower rate of adaptation to a high-fat diet has been shown to lead to weight gain over time, in particular with modern lifestyles incorporating a low level of physical activity (81). Flatt (82) proposed that subjects who continue to oxidize carbohydrate in the post-absorptive state deplete their endogenous carbohydrate stores, thereby stimulating food intake. This leads to increased food intake to replace these diminished carbohydrate stores and increased fat storage. Additionally, it has been hypothesized that a high postprandial fat oxidation may inhibit food intake (83). Through these mechanisms, inter-individual differences in substrate selection may have an impact on energy balance and may play a role in the development of obesity and subsequently T2DM. Additionally, a shift towards a greater postprandial fat oxidation may attenuate fat accumulation in non-adipose tissues, leading to improved insulin sensitivity (8).

### Incretins and weight management

**Figure 2:**
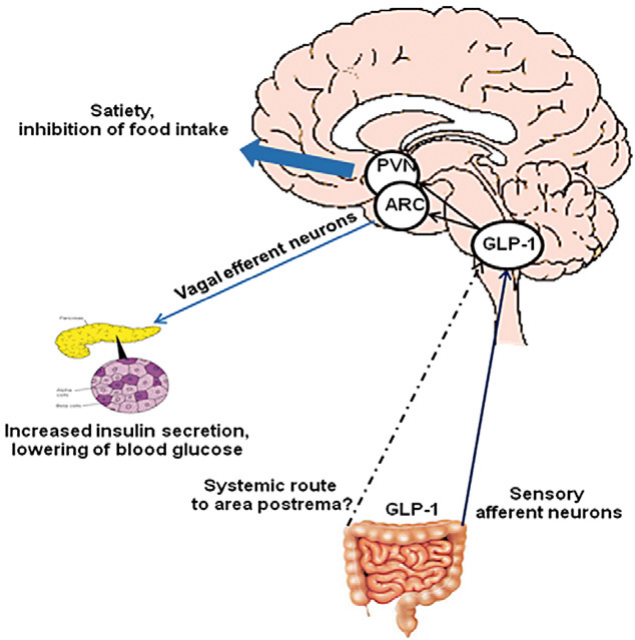
Glucagon-like peptide-1 (GLP-1) increase insulin secretion lowers blood glucose and inhibits appetite. The ingestion of food promotes the release of GLP-1 from L-cells in the intestine, which activates vagal afferents. Activated GLP-1 neurons of the nucleus of the solitary tract (NTS) project to the hypothalamic arcuate nucleus (ARC), to modulate vagal motor outflow to the pancreas and other tissues not depicted, increasing insulin secretion from the β-cells in states of hyperglycaemia and suppresses glucagon from the α-cells, leading to lowering of blood glucose. Systemic GLP-1 may also access the brain via leaks in the blood–brain barrier such as the subfornical organ and the area postrema, as demonstrated to occur in rats. In the brain, release of GLP-1 within the NTS and from projections of GLP-1 neurons to the paraventricular neurons (PVN) leads to GLP-1-R activation, which promotes satiety and anorexia. Besides from the actions depicted in the picture GLP-1 has numerous other actions including slowing gastric emptying and thereby flattening glucose excursions, as well as cardiac effects. Adapted from Torekov *et al*., Obesity Reviews 2011 (429), and Williams, Endocrinology. 2009; 150: 2997–3001 (430).

The incretin effect is the amplification of insulin secretion when glucose is taken in orally as opposed to intravenously. It is due to the combined actions of the incretin hormones: GLP-1 and GIP (see also the role of incretins in the section ‘Prevention of diabetes and IR’). However, GLP-1 also inhibits appetite and food intake in animals and humans (84–86), and recent studies involving the GLP-1 receptor antagonist exendin 9-39 have shown that GLP-1 is a physiological regulator of appetite and food intake (87,88) (Fig. 2). GLP-1 is encoded by the glucagon gene (GCG), which also encodes oxyntomodulin, glucagon and GLP-2. GLP-1 is synthesized in L-cells of the small intestine and is released postprandially in proportion to EI. Carbohydrate and fat seem to be especially effective stimuli for GLP-1 secretion (89). Native GLP-1 has a short elimination half-life of 1–2 min because of dipeptidyl peptidase-4-mediated degradation.

The dose-dependent inhibition of appetite by GLP-1 has been demonstrated in humans in a meta-analysis (90). Clinical studies of GLP-1 mimetics (exenatide [91]–[96], liraglutide [97]–[102], albiglutide [103] and taspoglutide [104]) in overweight/obese patients with T2DM have demonstrated significant and durable weight losses. Recent studies with the long-acting GLP-1 analogue, liraglutide, demonstrated dose-dependent weight losses exceeding that obtained with the lipase inhibitor, orlistat, also in non-diabetic obese individuals (63). In obese individuals, endogenous GLP-1 responses to meal intake may be severely reduced (86), suggesting that decreased GLP-1 levels may contribute to the development of obesity, although its appetite-suppressive potency appears to be preserved.

The other incretin hormone, GIP, does not have effects on appetite and food intake but may have actions on the adipose tissues, which express the GIP receptor. Knockout of the GIP receptor prevents diet-induced obesity in mice (105,106), and GIP may promote adipose tissue blood flow and uptake of fatty acids and their incorporation in triglycerides (TGs) (107,108). In contrast to GLP-1, meal-stimulated GIP levels are frequently elevated in obese individuals (109).

### Nutrients affecting incretin secretion and postprandial glycaemia

Because of the augmented secretion of the incretin hormones and their effects on glucose-induced insulin responses, healthy individuals can consume from 25 to 100 g of glucose without dose-related increases in plasma glucose. The incretin effect ensures that up to 80% of ingested glucose (100 g) is removed from the circulation and therefore does not contribute to increased postprandial glycaemia (110). The incretin hormones, therefore, play a very important role in postprandial glycaemia. Up-regulation of the incretin effect in response to increasing oral glucose loads seems to be crucial for controlling glucose excursions in healthy subjects. Patients with T2DM are characterized by an impaired capability to regulate their incretin effect, which may contribute to the exaggerated glucose excursions after oral ingestion of glucose in these patients (111).

In a recent study with the GLP-1 receptor antagonist exendin (9-39) amide, it was shown that GLP-1 released after an oral meal lowers postprandial glycaemia. The inhibition of glucagon release was a major determinant of the acute GLP-1 action in healthy subjects (112). Another study showed that the total GLP-1 AUC after a standardized breakfast was higher following an evening meal composed of barley bread compared with white wheat bread. Correspondingly, the glucose AUC after a standardized breakfast was higher following an evening meal composed of white wheat bread compared with barley bread (113). Thus, agents that enhance postprandial GLP-1 secretion are likely to result in decreased postprandial glycaemia. Because of the insulinotropic action of GLP-1 and GIP and the associated importance of the incretins in glucose homeostasis, much research has focused on the mechanisms of carbohydrate sensing by GLP-1- and GIP-producing cells. However, it is widely accepted that other macronutrients can also stimulate hormone release from these cells. Early studies indicated that protein was a relatively weak stimulus for GIP and GLP-1 in humans, although it probably contributed to the later phase of postprandial GLP-1 secretion (89). However, it has been shown that the GIP response is accentuated when milk proteins are delivered as peptide hydrolysates (114). Furthermore, daily intake of either a soy supplement or placebo (casein) significantly increases postprandial GIP response in the soy group compared with the casein group (115). Fat ingestion is widely considered a good stimulus for both GLP-1 and GIP secretion. It has been shown that GLP-1 responses were highest after an olive oil meal compared with a meal with saturated fat (butter) (116,117). Although direct exposure of the intestinal lumen to oleic acid did not always result in significant stimulation of GLP-1 secretion (118), the digestion of TGs to FFAs does seem a crucial step, as inhibition of pancreatic lipase prevents the stimulation of GIP and GLP-1 secretion. In the past few years, lipid-sensing mechanisms have been dominated by the idea that the newly discovered fatty-acid-sensitive G-protein-coupled receptors underlie enteroendocrine responses to a range of fatty acids and complex lipids. The mechanisms employed to sense the arrival of carbohydrate, fat and protein in the gut lumen have been investigated using organ perfusion techniques, primary epithelial cultures and cell line models. Furthermore, the current knowledge of the molecular machinery underlying nutrient sensing within GIP- and GLP-1-producing cells is covered in detail in the reviews by Reimann (119) and Parker *et al*. (120) in 2010.

Another way in which GLP-1 regulates postprandial glycaemia is by inhibiting gastric emptying. With high physiological doses of GLP-1 it is possible to completely arrest gastric emptying for a couple of hours, and in clinical studies with exenatide, postprandial glucose excursions are flattened considerably (121).

In summary, up-regulation of the incretin effect in response to increasing oral glucose loads seems to be crucial for controlling glucose excursions and thereby postprandial glycaemia. Furthermore, recent evidence points to a dual action of GLP-1, involved in both glucose homeostasis and appetite regulation (Fig. 2).

### Conclusions

Interest in the relationship between diet and health has increased the demand for functional foods (foods with specific beneficial properties beyond their basic nutritional contribution). In particular, the obesity epidemic has brought appetite control and nutrient partitioning to the forefront of functions susceptible to modulation by foods and food ingredients. Consequently, functional foods for the control of appetite (and ultimately body weight) now constitute a major health goal and research objective (122). The belief that certain foods can help to control appetite has led to a huge interest in identifying those properties of foods that can influence food intake with knock-on benefits for body weight control. These endeavours have resulted in the popularity of the concept of ‘functional foods for satiety and weight management’.

Some studies suggest that diets comprising foods that elicit a lower postprandial glucose response may be useful as part of an overall strategy for combating obesity, but the evidence for a role of postprandial glucose *per se* in these effects is considerably weaker. Diets based on foods that produce lower postprandial blood glucose responses (i.e. LGI foods) reduce the risk of developing diabetes (123), CVD (124) and certain cancers (125) while improving insulin sensitivity, blood glucose control and blood lipid profiles (126,127). There is also some evidence that LGI diets stimulate fat oxidation at the expense of carbohydrate oxidation (8) and reduce body fat deposition (8,128). The mechanisms relating glycaemic response to the regulation of body weight have been examined in controlled feeding studies (129) and involve acute hormonal and metabolic changes that may decrease hunger and EI. The biological system underlying appetite control is becoming better understood and involves close links between peripheral physiology, metabolism and brain processes. It also embodies mechanisms that are potential targets for foods designed to influence satiety. Of particular interest, for example, are gastro-intestinal peptides that are released following food consumption and involved in the control of appetite and satiety (cholecystokinin [CCK], GLP-1, peptide YY [PYY] and ghrelin).

Although some short-term studies suggest that LGI carbohydrates suppress hunger more effectively than high-GI carbohydrates, there is no strong mechanistic basis for normal variation in postprandial blood glucose *per se* (i.e. differences in blood glucose seen between typical high- vs. LGI treatments) to affect appetite (34–36) and ultimately body weight (38–41,43). Further, the known satiating effect of insulin would generally argue for more satiating effects of high-GI treatments and the studies where nutritional composition is carefully controlled also support this view. There are currently little data on the effects of GI on body weight. Hence, there is a clear need for more long-term well-designed human studies to evaluate how high- and LGI diets (containing foods of the same palatability, volume, energy density, macronutrient composition and fibre content) affect EI, body composition and body weight for biologically significant periods of time. Indeed, recently published (2010) opinions on dietary reference values (DRVs) for carbohydrates, dietary fibre, fats and water adopted by EFSA's Panel on Dietetic Products, Nutrition and Allergies (130) include the conclusion that evidence is still inconclusive on the role of GI and GL in maintaining weight and preventing diet-related diseases. In EFSA's opinion, the currently available long-term studies do not allow a reliable conclusion about the regulation of EI and body weight because the food constituents, carbohydrates that induce a low glycaemic response and those with an LGI are not sufficiently characterized. This is because the GI of a carbohydrate-containing food depends on several factors other than the amount of available (glycaemic) carbohydrates present. Such factors include the amount and type of dietary fibre, amount of dietary fat, physical properties, mode of preparation as well as individual factors (e.g. biological variation, rates and extent of digestion and absorption). In this respect, more studies that specifically manipulate postprandial glucose while quantifying the effects of this on satiety/appetite and body weight are required. In conclusion, unless a universal relationship between blood glucose responses and satiety/appetite or other weight outcomes is demonstrated, LGI diets or low glycaemic responses will not assure enhanced satiety or weight management.

## Postprandial glucose in relation to oxidative stress and inflammation^2^

### Introduction

Epidemiological studies support the evidence that postprandial (2 h) glucose is an independent predictor of CVD and diabetes. Studies indicate that postprandial hyperglycaemia contributes up to 70% of total daytime hyperglycaemia. In T2DM, a positive correlation with HbA1c concentration was reported for postprandial and preprandial glucose concentrations (131). The contribution of postprandial glucose in HbA1c concentration predominates in patients with fairly good control, whereas the contribution of fasting hyperglycaemia increases as glycaemic control worsens (132). Many studies have reported a stronger association of CVD risk and postprandial or postload glucose than for CVD risk and HbA1c or mean blood glucose. However, HbA1c and mean blood glucose show stronger associations with CVD risk factors than do postprandial glycaemia or glucose variability in people with diabetes (the A1C-Derived Average Glucose [ADAG] study) (133). Also, specifically targeting postprandial glucose by using prandial insulin or meglitinides has not unequivocally resulted in improvement of the CVD risk profile (134,135). Importantly, there appears to be no glycaemic threshold for reduction of either microvascular or macrovascular complications. The progressive relationship between plasma glucose and cardiovascular risk extends well below the diabetic threshold (136,137). In this paragraph, both chronic and acute hyperglycaemia in relation to oxidative stress and inflammation will be addressed. Acute glucose variations from peaks to nadirs include postprandial glucose excursions that can be described by two components. The first component, the duration of the postprandial glucose increment, is a major contributor to chronic sustained hyperglycaemia, while the second component, the magnitude of the postprandial rise, is more a reflection of glucose variability. It is difficult to discriminate between the contributions of these two components of dysglycaemia. It seems that both contribute to the two main mechanisms that lead to diabetic and cardiovascular complications, namely excessive protein glycation and activation of oxidative stress.

Hyperglycaemia, elevated TGs/fatty acids and hyperinsulinaemia, which are particularly pronounced in the postprandial state in individuals with IR and T2DM, are abnormalities that often cluster, which makes it more difficult to tease out and to study single effects. There are indications that they may have distinct mechanisms of action and, when present together, act synergistically to induce oxidative stress, protein kinase C (PKC) activation and advanced glycated end-product receptor (RAGE) activation (138,139). Collectively, these derangements promote inflammation, impaired endothelial function and vascular disease (140,141). Besides, oxidative stress is not only associated with the complications of obesity or diabetes but has also been linked to IR *in vitro* and *in vivo*, both major features of T2DM (142). To obtain a more detailed understanding of the relationship between hyperglycaemia (and related hyperlipidaemia and hyperinsulinaemia) and oxidative stress and inflammatory responses, insight into the underlying mechanisms might be of crucial importance.

### Elevated glucose induces oxidative stress and inflammation

There is now cogent evidence for a deleterious effect of sustained chronic hyperglycaemia that results in excessive protein glycation and generation of oxidative stress. Notably, it is important to distinguish between hyperglycaemia within the normal physiological range in normal glucose-tolerant subjects and more pronounced excursions in the impaired glucose-tolerant or diabetic state (143). Intravenous glucose infusion in obese glucose-tolerant subjects promotes oxidative stress, and it has been shown that caloric restriction in obese subjects for a brief period of 4 weeks leads to a significant reduction of oxidative stress (144). Also, glucose intake induces oxidative stress and inflammation at cellular and molecular levels and, under certain conditions, an acute increase in blood glucose is associated with an acute inflammatory response. *In vivo*, oral glucose challenges increase plasma levels of adhesion factors (ICAM-1, VCAM-1 and e-selectin), cytokines (interleukin [IL]-6, tumour necrosis factor alpha [TNFalpha]) and chemokines (IL-8) in healthy volunteers (145). Finally, the consumption of a high-glycaemic meal increases oxidative stress and reduces antioxidant defences, with the increase being significantly greater with higher levels of hyperglycaemia in young healthy volunteers (146).

Thus, as indicated above, glycaemic excursions within the physiological range in healthy volunteers induce physiologically significant effects on endothelial function, oxidative stress and immune activation. Nevertheless, in normal glucose-tolerant subjects these inflammatory responses normalized within 2–3 h, while in obese impaired glucose-tolerant and type 2 diabetic subjects, glucose-induced inflammation was stronger or lasted longer, suggesting a role for hyperglycaemia in immune activation (147–150). Also in diabetic patients, after oral glucose and after meals, reactive oxygen species (ROS) and/or LDL oxidation was increased, indicating hyperglycaemia-induced activation of oxidative stress (151). Several markers have been used to assess oxidative stress and the antioxidant status in patients with diabetes, including ox-LDL and malondialdehyde (152). The short plasma half-life of these markers is one of the limiting factors for the assessment of oxidative stress in plasma samples. Thus, when available, urinary measures provide a more reliable determination of the activation of oxidative stress than plasma measurements. From several studies, both *in vitro* and *in vivo*, there is consistent evidence that hyperglycaemia is associated with an increased urinary excretion rate of the oxidative stress marker 8-iso-prostaglandin F2alpha. Ceriello (153) showed that the production of free radicals was increased in the postprandial period and that this increment was proportional to the magnitude of postprandial glucose excursions. Moreover, reductions of postmeal glucose excursions induced by a premeal bolus of a rapid insulin analogue resulted in decrements in glycaemia and nitrotyrosine, a metabolite derived from nitrosamine stress responses. Overall, numerous studies support the hypothesis of a causal relationship between hyperglycaemia and oxidative stress.

This seems consistent with findings that the metabolic syndrome may support peripheral inflammation by sensitizing leukocytes to up-regulate pro-inflammatory markers in response to glucose, which in turn increases the risk for T2DM and CVD (154).

### Glucose fluctuations and the activation of oxidative stress

The role of glucose variability from peaks to nadirs is less well documented, but there are indications that postprandial swings in glucose levels may increase oxidative stress. *In vitro* studies do show a relationship between glycaemic variability and oxidative stress-induced apoptosis and renal cell proliferation in cultures human or rat cells, and these findings are confirmed in an animal study (as reviewed by Siegelaar *et al*. [155]).

Experimentally induced acute oscillations in glycaemia resulted in greater increments in plasma concentrations of some pro-inflammatory cytokines, such as IL-6, IL-18 and TNFalpha, than chronic hyperglycaemia in both healthy and glucose-intolerant subjects (148). Also, glucose at two different levels (10 and 15 mM) resulted in a concentration-dependent induction of both endothelial dysfunction and oxidative stress in both normal and T2DM patients, which seemed independent of the total amount of glucose to which subjects are exposed. Vitamin C infusion was shown to reverse hyperglycaemia-induced oxidative stress endothelial dysfunction in normal subjects but not in diabetic subjects. This suggests different underlying mechanims in the two groups: one due to the actual level of glycaemia and one due to the long-lasting damage induced in the endothelial cells by chronic hyperglycaemia (156). With respect to the design of the latter studies, the number of consecutive periods with alternating degrees of glycaemia that are necessary to reliably assess glycaemic variability can be debated. However, it may not be surprising that these artificial repetitive elevations of blood glucose in normoglycaemic individuals, that equalled glycaemic excursions in T2DM patients, resulted in similar induction of oxidative stress since blood glucose changes within the normal range, which occur in the postprandial state in normoglycaemic subjects, have been shown to raise markers of oxidative stress (see the section ‘Elevated glucose induces oxidative stress and inflammation’).

The results from cross-sectional studies investigating the relationship between glucose variability, obtained from continuous glucose monitoring (CGM), and oxidative stress are, however, less consistent (155). Monnier *et al*. measured 24 h urinary excretion of 8-iso-prostagladins F2alpha (8-iso-PGF2alpha), an indicator of free radical production, and performed CGM in T2DM patients. There was a linear correlation between increased free radical production and the magnitude of glucose fluctuations (expressed as MAGE), but not with the mean 24-h glucose concentration, fasting plasma glucose or even HbA1c. 8-iso-PGF2alpha concentrations were four times higher in patients with the greatest as compared with patients having the lowest glycaemic variability (157,158). Furthermore, exposure of lean non-diabetic subjects to the same magnitude of glycaemic excursions, as reported by Monnier *et al*., doubled circulating nitrotyrosine concentrations (159).

However, a second study by Wentholt *et al*. (160) addressing the same relationship in type 1 diabetic subjects could not confirm the findings of Monnier. According to the authors, this discrepancy was due to methodological factors and differences in populations. Indeed, another study using tandem mass spectrometry could not reproduce a relationship between glucose variability and oxidative stress (161). Also, a crossover trial comparing the effect of a basal insulin regimen and a meal-time insulin regimen on glucose variability and oxidative stress in T2DM patients could not detect any differences in oxidative stress or any correlation between glucose variability and 24-h excretion rates of 8-iso-PGF2alpha (162). Thus, to date, the relationship between glucose variability found in cell cultures and animal studies has not been consistently reproduced in human studies. Differences in methodology used for oxidative stress quantification, as well as differences in the duration and frequency of periods with alternating glycaemia, and populations may be possible explanations.

It has been hypothesized that repeated late postprandial glucose concentrations below the fasting level induced by carbohydrates with a high-glycaemic or insulinaemic index could activate counter-regulatory stress hormones such as cortisol and catecholamines. These hormones restore fasting glucose concentrations and increase non-esterified fatty acid concentrations but may also be involved in the development of IR and metabolic complications. This is supported by the finding that the modification of carbohydrate intake by replacing cereal products (oat and wheat bread) with rye bread and pasta, which are characterized by a low postprandial insulin response, differentially modulates the mRNA expression of inflammatory markers in both adipose tissue and circulating concentrations of serum in men and women with metabolic syndrome, even in the absence of weight loss (163). Indeed, after consuming the wheat vs. the rye bread, glucose levels dropped below fasting levels (average 6.3 ± 0.7 mmol L^−1^) 3 h after meal ingestion, whereas FFA increased concomitant to slightly rising levels of inflammatory markers and epinephrine (163). These data suggest that beside glycaemic variability, the pattern of insulinaemic response may evoke differential inflammatory and oxidative stress responses as well as varying counterregulatory hormonal changes, all of which may impact on diabetes and cardiovascular risk in the long-term.

### Tools for measuring glycaemic variability

Several approaches have been proposed, but for two main reasons, all fail to provide a complete representation of glucose oscillations. First, glycaemic variability is a complex phenomenon that includes both intraday and interday variability. Thus, glycaemic variability is the composite of vertical and horizontal components. The second reason why it is a complex phenomenon is that the glycaemic response is a combination of minor and major glucose fluctuations. It is still difficult to know whether the deleterious effects of glycaemic variability, such as activation of oxidative stress, are only triggered by major glucose swings or by all glucose oscillations, including the minor ones. For this reason, it is not surprising that several approaches have been developed for quantifying glycaemic variations. From a statistical point of view, the SD around the mean glucose value appears as the ‘gold standard’ for quantifying glycaemic variability. By contrast, the MAGE index is probably more appropriate for selecting the major glucose swings that are calculated as the arithmetic mean of differences between peaks and nadirs, provided that the differences are greater than the SD around the mean values. Furthermore, calculating the MAGE index requires CGM, which has the advantage of detecting all upward and downward acute glucose fluctuations (157). Finally, the mean of daily differences currently remains the sole index for estimating interday glycaemic variability. This parameter is calculated as the mean of absolute differences between glucose values at the same time on two consecutive days.

## Underlying mechanisms

It has been suggested that nitric oxide (NO), a potent vasodilatator, may play a key role in hyperglycaemia-induced oxidative stress and related effects on inflammation, endothelial function and insulin sensitivity. Hyperglycaemia may induce NO oxidation (164), thereby leading to reduced NO concentrations, which then lead to impairments in vasodilation. Indeed, alloxan diabetic rats are observed to have reduced NO concentrations in blood (165), and in human subjects, acute hyperglycaemia attenuated endothelium-dependent vasodilatation (166). The fact that such a decrease can be prevented by supplying various antioxidants such as vitamin C, E and alpha-lipoic acid (167–169) or L-ariginine, the precursor of NO (165,170), suggests that increased NO oxidation, and the related NO, reduces impact on the vascular epithelial cells. Indeed, restoration of NO availability results in the normalization of endothelial function as well as insulin sensitivity (168,169). Secondly, it has been hypothesized that acute hyperglycaemia may induce a depletion of vitamin C from cells because vitamin C and glucose share a common transport system and oxidative stress leads to use of intracellular vitamin C (171). Thirdly, elevated glucose, elevated lipids and hyperinsulinaemia occur simultaneously, in particular in the postprandial state (141). As hypothesized by Evans (167,172), chronic elevation of hyperglycaemia and FFA cause oxidative stress along with the activation of stress-sensitive signalling pathways, like nuclear factor kB (NFkB), p38 MAPK and NH2-terminal Jun kinases/stress-activated protein kinases pathways. This happens along with the activation of the advanced glycosylation end-products (AGE) receptor (RAGE), PKC and sorbitol stress pathways. Activation of these pathways, in turn, worsens both insulin action and secretion, leading to overt T2DM and playing a key role in the late complications of T1DM and T2DM. Glucose causes oxidative stress because of an increased production of ROS, non-enzymatic glycation of proteins and glucose auto-oxidation. Elevated FFA can cause oxidative stress because of increased mitochondrial uncoupling and ß-oxidation, the latter leading to an increased production of ROS and to an activation of stress-sensitive signalling pathways, which in turn impair insulin secretion and action.

It is important to note the fact that in human endothelial cells, *in vitro* elevation of extracellular D-glucose is not sufficient to promote a deleterious effect on vasculature but that a pro-inflammatory stimulus such as IL-1β is necessary. These data support a synergistic action of hyperglycaemia and inflammation and highlight the pivotal role of a pro-inflammatory environment in diabetes as a critical factor conditioning the early pro-atherosclerotic actions of hyperglycaemia (173). The precise mechanisms through which glucose variability may be worse than constant glucose levels remains incompletely defined. Studies in cells and animals have shown that in oscillatory pathways involving PKC, NADPH (174), inducible NO synthase (164) or inflammatory marker responses are more activated as a result of intermittent glucose than by constant high glucose levels (175,176).

## Role of diet

Studies of GI and dietary GL provide further support that postprandial hyperglycaemia increases the risk of CVD and T2DM. Hu *et al*. (177) observed a stepwise relationship between dietary GI and oxidative stress markers in healthy adults. Furthermore, high-GI carbohydrates increase NFkB activation and NFkB binding in mononuclear cells of young, lean healthy subjects (146). Diets low in GL and high in whole grains may have a protective effect against systemic inflammation in diabetic patients, as reviewed elsewhere (178). Consistent with this, epidemiological studies show an inverse relationship between dietary fibre and C-reactive protein (CRP) levels. Both the DASH diet (naturally high in fibre, i.e., 30 g fibre d^−1^) and a fibre-supplemented usual diet (30 g psyllium fibre d^−1^) decreased CRP concentration, but this finding was only significant in lean normotensive subjects in both intervention arms (179). On the other hand, a high-carbohydrate, low-fat diet with a relatively high dietary fibre and complex carbohydrate content, within the context of a lifestyle intervention programme, has been shown to reduce diabetes incidence in the long-term by 50% (5–7).

The prominent role of type of carbohydrate is also illustrated in studies showing that dietary carbohydrate modification, i.e. an oat/wheat/potato diet, upregulated 62 genes related to stress, cytokine-chemokine-mediated immunity and the IL pathway compared with a rye–pasta diet (180). As discussed above, these differences in inflammatory response may be related to differences in the early insulin response and the resultant late hypoglycaemia in the oat/wheat/potato diet group.

A noteworthy development is the increasing interest in the role of non-digestible carbohydrates in the manipulation of gut microbiota, thereby affecting energy harvesting from the gut, obesity, incretin levels, glucose intolerance and the pro-inflammatory response. Animal studies have proposed mechanisms by which a microbiota-related signal may link a high-fat diet to the development of obesity and IR by promoting a low-grade inflammatory state by increasing gut and systemic bacterial lipopolysaccharides derived from gram-negative bacteria (partly mediated through an increased leakage from the gut) (181). Currently, there is an urgent need for confirmation of these effects in humans using targeted intervention studies. Thus, appropriate dietary measures may have the potential to reduce postprandial glucose and postprandial glucose variability.

## Conclusion

The progressive relationship between plasma glucose and CVD, and the finding that there appears to be no glycaemic threshold for the reduction of either micro- or macrovascular complications (182), indicates that early treatment/prevention of hyperglycaemia is important. Furthermore, because there is no clear threshold for the adverse health effects, prevention of hyperglycaemia is of utmost importance in diabetes as well as in healthy volunteers and high-risk subjects. The goal should be to achieve a glycaemic status as close to normal as is safely possible in all three measures of glycaemic control: fasting glucose, HbA1c and postprandial glucose. Finally, large lifestyle intervention trials have shown that appropriate dietary measures are of benefit in reducing postprandial glucose and postprandial glucose variability.

## Prevention of diabetes and IR^3^

### Introduction

T2DM is mainly due to an association between a defect in insulin secretion and IR. It leads to alteration of postprandial glycaemia with both higher glucose levels and sustained hyperglycaemia. Better glycaemic control, as assessed by glycated haemoglobin (HbA1c), should be obtained by control of both fasting plasma glucose values (183) and post-meal glycaemia as recently highlighted (184). Actual recommendations for diabetes care are that postprandial glycaemia should not exceed 140 mg dL^−1^, 2 h after a meal. Thus, strategies targeting postprandial glycaemia are being developed, including both dietary approaches to reduce dietary GL and pharmacological approaches involving the use of specific drugs (α glucosidase inhibitors, glinides, GLP-1 derivatives, rapid-acting insulin) (182). However, whether or not reduction of postprandial glycaemia will reduce the risk of diabetes in healthy or at-risk subjects remains controversial.

In this section, the role of postprandial glycaemia in the prevention of diabetes in healthy or at-risk subjects with an IGT will be addressed. Years that precede the development of T2DM are characterized by a progressive decline in both insulin action and defects in the early phase of the insulin secretion, with losses of postprandial glucose regulation occurring before overt diabetes (185). To prevent diabetes, modulation of postprandial glycaemia should interfere either with insulin sensitivity and/or with insulin secretion. Such interactions can be speculated in accordance with the concept of glucotoxicity and glucolipotoxicity and their potential action on both insulin secretion and insulin sensitivity. We will review epidemiological data and intervention studies. For shorter-term interventions, attention will be paid to actions on either insulin sensitivity or secretion. Data on the link between postprandial glycaemia and risk of diabetes could only be obtained in long-term studies. With respect to the latter studies, we will only report interventions specifically targeting postprandial glucose response, which are mainly drug interventions as lifestyle interventions are not thought to have a specific effect on postprandial glucose. Finally, potential underlying mechanisms will be considered.

### Observational data in cohorts

The link between diet and the risk of diabetes has been studied in numerous prospective studies in large cohorts. As carbohydrate is the main dietary component affecting insulin secretion and postprandial glycaemia, attention has been focused on this macronutrient. Both the amount and the type of carbohydrate have an effect on postprandial glycaemia and insulin secretion, and GI and GL are indicators of postprandial glucose response. Decreased risk of diabetes has been shown to be associated with an LGI diet. Willett *et al*. (10) have used data from a large prospective epidemiological study of women (Nurses' Health Study) as well as data from a large prospective epidemiological study of men (Health Professional's Follow-Up Study) to verify the hypothesis that high dietary GI and/or GL increases the risk of T2DM. They showed that women in the highest quintile of GL had a 40% greater risk of T2DM as compared with women in the lowest quintile. Addtionally, for men in the extreme upper categories of GL and lower categories of cereal fibre intake, the relative risk (RR) of diabetes was RR = 2.17 (high glycaemic load [>188] and low cereal fiber intake [<3.2]). In the Nurses' Health Study, Liu *et al*. (186) observed that fasting triacylglycerol concentrations were nearly twofold higher among women in the highest GL quintile than among those in the lowest quintile. Barclay *et al*. (187) used meta-analysis techniques to evaluate the association between GI and/or GL and the occurrence of several chronic diseases. For this purpose, these authors selected (among 274 potentially relevant references) 37 original prospective observational cohort studies from which 8 were devoted to the risk of diabetes. Of these 8 studies, 6 were in favour of an association and 2 were not. Based on comparisons between subjects ingesting diets with high or LGI and/or GL, the meta-analysis showed that a high GI or GL independently increased the risk of T2DM (GI RR = 1.4, GL RR = 1.27).

Taken together, these observational data suggest that replacing high-GI with LGI carbohydrates reduces the risk of metabolic disturbances and T2DM. Nevertheless, inconclusive results were also found in some studies. Methodological issues might explain the failure to show the association between LGI and/or GL and the development of T2DM. For example, in the large prospective cohort of 35,988 older Iowa women studied by Meyer *et al*. (188), the GI or GL was not significantly related to the risk of T2DM. This could have been due in part to the use of a single measure of dietary intake and/or the fact that the presence of T2DM was based on self-report without confirmatory information.

Decreased diabetes risk has also been shown to be associated with whole-grain (189) and green leafy vegetable intake (190) and even nut consumption (191). From these studies, it is not possible to know if these diets influence postprandial glycaemia, but we have several arguments to think so. The RR of T2DM for the highest as compared with the lowest quintile of whole-grain intake was 0.63 in the Nurses' Health Study I and 0.68 in the Nurses' Health Study II. Vegetables, unrefined whole grain and bran products are complex substances that contain both soluble and insoluble fibre. Soluble viscous fibres reduce postprandial glucose responses and cereral insoluble fibres have been shown to improve insulin sensitivity in overweight and obese subjects using a euglycaemic hyperinsulinic clamp over 3 d (192). Moreover, after 1 d of consumption, the postprandial glycaemic response was significantly decreased with no change in peak glucose but with a decrease in AUC. It was associated with an increase in early insulin and GIP secretion (193). Thus, fibre consumption seems to be associated with a decrease in postprandial glucose AUC. This effect should be at least partly responsible for the reduced risk of diabetes found in whole-grain consumers. Finally, in the Nurses' Health Study, nut consumption has been shown to be associated with a decreased risk of T2DM (RR 0.78 for more consumption than five times per week compared with never) (191), and nuts have been shown to decrease the postprandial glucose response in a dose-dependent manner (decreased AUC and peak) (194). Thus, most observational studies show that diets decreasing postprandial glycaemia are associated with a lower risk of diabetes.

### Long-term intervention studies

Acarbose is an α-glucosidase inhibitor that reduces postprandial glycaemia by decreasing glucose absorption. The STOP-NIDDM study randomly allocated 714 participants with IGT to 100 mg of acarbose and 715 participants to placebo. The risk of progression to diabetes over 3.3 years was reduced by 25% with acarbose. Furthermore, acarbose increased the probability that IGT would revert to normal glucose tolerance over time (195). On the contrary, in the Early Diabetes Prevention Program, 219 subjects with early diabetes (HbA1C = 6.3%) were randomly assigned to 100 mg of acarbose or placebo with a follow-up of 5 years. Despite a decrease in postprandial glycaemia (post-peak and AUC) under acarbose, there was no difference in the cumulative incidence of fasting hyperglycaemia. However, a post hoc analysis showed that in subjects with initial fasting glycaemia lower than 126 mg dL^−1^, acarbose reduced the rate of development of fasting hyperglycaemia. This suggests that the lack of observed effect during the study was due to the inclusion of subjects with a too far advanced stage of glucose metabolism abnormalities. A meta-analysis of the α glucosidase inhibitor intervention studies included five trials with IGT or at-risk subjects and confirms the reduced risk of diabetes by, on average, 20% (196).

The NAVIGATOR study, a double-blind, randomized clinical trial, assigned 9,306 participants with IGT or cardiovascular risk factors to receive nateglinide (up to 60 mg three times daily) or placebo, in a 2-by-2 factorial design with nateglinide or placebo, in addition to participation in a lifestyle modification programme (197). Nateglinide is a short-acting insulin secretagogue. It reduces postprandial glucose by enhancing insulin secretion. However, this treatment was unable to reduce the incidence of diabetes. The difference between this study and the acarbose trials may be due to the difference in action of the two drugs, i.e. the induced insulin secretion by nateglinide compared with the reduced blood glucose response through decreasing glucose absorption with acarbose.

The human GLP-1 analogues are now currently used for the treatment of T2DM. These analogues decrease HbA1c by improving insulin secretion through restoring the first phase of insulin secretion, acting mostly on postprandial glycaemia. The GLP-1 analogue liraglutide has been given to a large group of overweight/obese participants (4% with T2DM, 33% with an impaired glucose metabolism, 63% healthy volunteers) for 20 weeks (63). During therapy (which led to significant and durable weight losses) glucose tolerance was normalized in 90% of the subjects. Insulin sensitivity assessed by homeostatic model assessment (HOMA) index was unchanged, whereas β cell function was improved (HOMA β). Longer-term studies are required, but these initial results appear very promising. Differences in the profile of insulin secretion between glinides and GLP-1 agonists could possibly explain the differences between the results of the latter study and the NAVIGATOR study. Thus, long-term interventions with drugs targeting postprandial glycaemia could be efficient in reducing the risk of diabetes, but the results are dependent upon both the mechanism by which the postprandial glycaemia is decreased and the glucometabolic status of the subjects, intervention being less efficient in subjects with a too far advanced stage of abnormalities in glucose metabolism (196).

### Short-term intervention studies

For short-term intervention studies, it is important to examine intermediate risk markers for T2DM such as the postprandial glycaemic response in relation to insulin sensitivity or insulin secretion. Maintenance of a normal plasma glucose concentration requires precise balance between glucose utilization, its endogenous production mainly by the liver (glycogenolysis and neoglucogenesis) and its exogenous absorption at the intestinal level. The rise in postprandial glycaemia will depend on the quality of the ingested carbohydrate, on its interaction with other compounds, as well as on the ability of the participant to clear the glucose by insulin-induced glucose utilization. The type of carbohydrate and its digestibility, the quantity of carbohydrates as compared with other macronutrients such as lipids, proteins and fibres are all confounding parameters that can dramatically modify the post-meal metabolic response. Thus, we aim to review the influence of the type of carbohydrate on the postprandial glucose response and the metabolic consequences on insulin sensitivity and secretion. Unfortunately, in several intervention studies, the information on the characteristics of the postprandial glucose response are not detailed and sometimes not even controlled, and the diet has mostly been only characterized by GI.

#### Influence of the type of carbohydrate

Intervention studies examining the influence of carbohydrate on IR and diabetes have compared different types of carbohydrates, mainly SUC vs. fructose vs. starch. These studies are often short-term, but the time course of glycaemia, insulinaemia and TGs are often described. After ingestion of fructose, postprandial glucose is reduced compared with after SUC. Nevertheless, fructose may stimulate liver lipogenesis that may increase triacylglycerol concentrations. These effects are dose dependent and low intake of fructose (less than 50 g d^−1^) could be beneficial by decreasing Hba1c and glycaemic reponse, whereas a high amount (more than 90 g d^−1^) may be deleterious with decreased insulin sensitivity, an increased liver fat content and increased TGs (51,52,198,199).

Several acute intervention studies in healthy participants show that the preservation of starch in its original state, which is low gelatinized (i.e. slowly digested), induces a lower glycaemic response and a lower insulin demand during the post-meal period compared with several processed starchy foods in which starch is highly gelatinized (rapidly digested) (22,200–203). The mechanism by which less gelatinized starch leads to a lower glycaemic and insulin response has been identified using isotope-labelling methods. The authors demonstrated that the consumption of a cereal product with a high content of slowly available glucose induced a lower appearance rate of exogenous glucose in the bloodstream during the postprandial period (204).

Intervention studies where postprandial excursion of glucose is followed are mainly based on GI manipulations. The introduction of LGI foods into a daily diet is able to decrease the global hyperglycaemia level across the whole day in healthy subjects, with lower hyperglycaemic peaks after meals and lower glycaemia during post-absorptive phases, which may be most pronounced during the night (205,206). However, discrepancies are observed in these results and may begin with the definition of low- vs. high-glycaemic diets. Foods that do not contain carbohydrates or that do no contain enough carbohydrates to raise blood glucose should not be included in the estimation of the GI value of diets (207), and a large range of values is obtained for the difference in GI between the high- vs. the LGI diets. Moreover, despite a good selection of food products belonging to the low- and high-GI diets, some studies failed to show differences in postprandial glucose kinetics (56). This was also the case for the study by Black *et al*. (208), who showed no differences in glycaemic profiles obtained using CGM during 25% vs. 10% SUC diets in healthy participants. Finally, numerous studies investigating different GI diets did not report the effect of their intervention on postprandial glycaemia.

#### Short-term intervention studies

Tables 3–5 report the 21 different intervention studies, 11 of which were able to show an effect either on insulin sensitivity or on insulin secretion, whereas 10 were not. Only 9 studies reported postprandial glycaemic parameters. Four of these studies showed no differences in postprandial glycaemia between the two intervention groups. We therefore decided to comment only on the five papers in which postprandial glycaemia was mentioned and was significantly different between groups.

**Table 3:** Studies showing an effect on insulin sensitivity

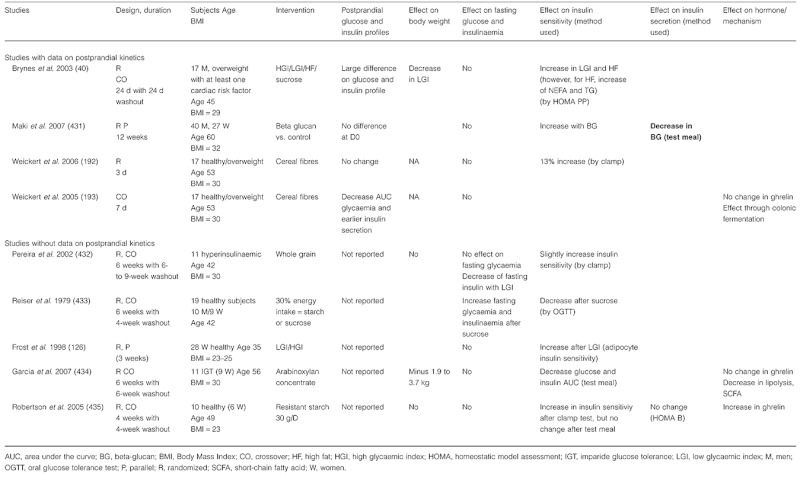

**Table 4:** Studies showing an effect on insulin secretion

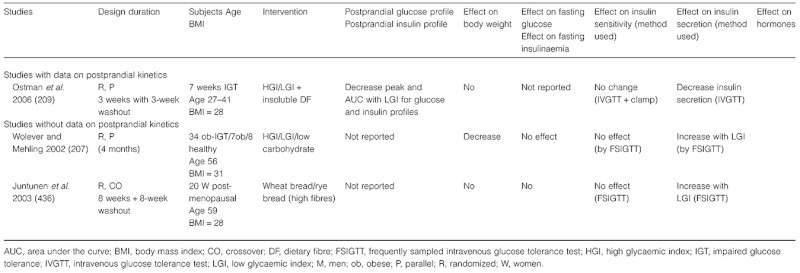

**Table 5:** Studies showing no effect on insulin secretion

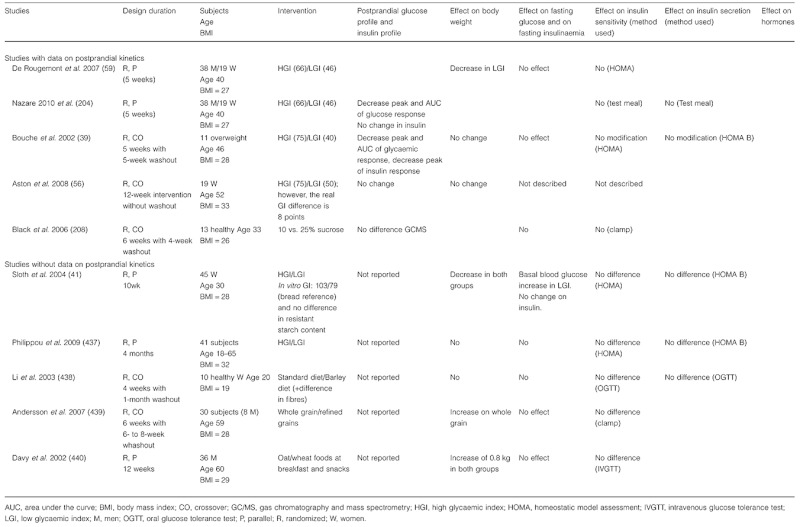

In overweight participants, a similar decrease in postprandial glucose and postprandial insulin responses was found after 4 and 5 weeks of consuming an LGI diet compared with a relatively high-GI diet, which was associated with a slight weight or fat mass reduction (39,40), respectively. Brynes *et al*. also showed an improvement in insulin sensitivity (as estimated by HOMA) after repeated lower glucose excursions, whereas Bouché*et al*. showed no difference. The difference in the effect on insulin sensitivity may be related to the magnitude of the blood glucose response difference between both GI diets. During the experimental days, in the study by Bouché*and collegues*, mean blood glucose peaks after breakfast were 7.7 mmol L^−1^ and 6.6 mmol L^−1^ for high- vs. LGI diets after 5 weeks of intervention, while in the study by Brynes *and colleagues*, mean blood glucose peaks after breakfast were 8.9 mmol L^−1^ and around 7.5 mmol L^−1^ after 4 weeks of intervention. Moreover, in the study by Nazare *and co-workers*, the authors could not find any improvement in insulin sensitivity after 5 weeks of an LGI diet compared with a high-GI diet in overweight subjects (204). One of the explanations may be that the magnitude of difference in glucose responses was too low (less than 1 mmol L^−1^ difference at the mean blood glucose peaks after breakfast between both diets) and that there were no significant differences in insulin responses between both test breakfasts and lunch during the experimental days. Another explanation would be that the Nazare sample was younger and had lower basal glycaemia, insulinaemia and total cholesterol compared with the Brynes and Bouché studies. In another study on seven overweight women with IGT, the authors evaluated modest dietary changes by modulating the GI characteristics and the dietary fibre content of bread, eaten during 3 weeks as part of their usual diet (209). The LGI/high-fibre bread diet led to a reduced insulinaemic response of 35% during the first hour after euglycaemic-hyperinsulinaemic clamp but had no impact on glucose response (209).

Additonally, in the study of Bouché and colleagues the authors showed that fasting total cholesterol and Apo Lipoprotein B decreased after an LGI diet period of 5 weeks (39). The effect of a reduced postprandial glucose response on fat metabolism in obese non-diabetic subjects was found elsewhere. During the same intervention period (as in Bouché*et al*.), De Rougemont *et al*. showed a 10% decrease in total cholesterol, a 9% decrease in LDL cholesterol and a 10% decrease in the ratio of LDL : high-density lipoprotein (HDL)-chlolesterol, after an LGI diet compared with after a high-GI diet (59). Harbis *et al*. demonstrated that a meal rich in slowly digestible carbohydrates induced low glycaemic and insulinaemic responses, and reduced the postprandial accumulation of both hepatically and intestinally derived triacylglycerol-rich lipoproteins in obese subjects with IR (210).

Livesey *et al*. (51,52) performed a systematic review of experimental data reported in intervention studies on the association between GI and markers of metabolic disorders through the use of meta-analysis and meta-regression models. Participants were either normal weight (16 studies), overweight (18 studies), obese (10 studies) or unclassified by weight (1 study). Study participants were either healthy, i.e. no evident diagnosis of disease (13 studies), or had IGT (2 studies) or had T1DM (7 studies), or T2DM (17 studies) or were at risk of primary CHD (4 studies) or secondary CHD (1 study) or had hyperlipidaemia (1 study). The analysis shows that diets with an LGI reduced both fasting blood glucose and glycated protein levels. These effects were greater in participants with poor fasting blood glucose control but were also observed for fasting capillary blood glucose levels slightly above 5 mM. Twelve studies reported an increase in insulin sensitivity in non-diabetic subjects on an LGI diet. Hence, diets with an LGI have a beneficial impact on some biological parameters such as fasting blood glucose, glycated protein control and insulin sensitivity in healthy subjects. Unfortunately, insufficient data on postprandial glucose profiles were available.

Finally, short-term intervention studies give no clear-cut results. The number of studies is too low, the studies are too heterogenous for the type of intervention and measurements of postprandial parameters are too scarce to be able to drive conclusions. More studies devoted to this question are required. However, the last meta-analysis showed a positive impact in the reduction of postprandial blood glucose on the prevention of diabetes (51,52). Furthermore, it seems that in order to evaluate the impact of postprandial hyperglycaemia on glucose and lipid metabolism, it is necessary to sufficiently manipulate the meals or diets to obtain a difference.

### Experimental arguments – mechanisms

The mechanisms by which modulation of postprandial glycaemia can interfere with insulin sensitivity and secretion and thus the risk of diabetes involve the concept of glucolipotoxicity. This concept has been largely illustrated on insulin secretion with experiment on pancreatic islet but has also been demonstrated in humans, particularly in T2DM.

The pathophysiology of IR has been more related to lipotoxicity than to glucotoxicity. IR is often characterized by systemic lipid overflow, leading to lipid accumulation in non-adipose tissues (ectopic fat accumulation), which is a strong risk marker for IR. Lipid uptake by muscle is largely dependent on the circulating FFA and TG concentrations. Glycaemia, insulinaemia, and TG and FFA levels are closely linked. Manipulation of postprandial glucose could be associated with either an increased insulin secretion (if decrease of glycaemia is induced by insulin secretion) or a decreased insulin secretion, as is usually the case with studies of LGI. An increase in insulin secretion in the short-term is associated with a decrease in TG and FFA concentrations and lipid oxidation that should be favourable for insulin sensitivity, as in the short-term this may reduce lipid overflow and reduce ectopic fat accumulation. However, subjects prone to T2DM have an increase in body weight and (dysfunctional) fat mass that leads to increased level of TGs and fatty acids. Recent studies have shown that an increased fat mass and the accompanying hyperinsulinaemia are inversely related to expression of lipoytic enzymes in adipose tissue (adipose triglyceride lipase, hormone sensitive lipase, etc.) and to whole body rate of appearance expressed per unit fat mass. Thus, there is mixed evidence for the notion that the obese insulin-resistant or diabetic state is associated with raised postprandial, diurnal or nocturnal FFA concentrations and more emphasis may be placed on raised TGs as a source of lipid overflow. Nevertheless, in diabetic subjects an increase in postprandial glycaemia will be associated with higher TGs and/or FFA levels as compared with normal subjects. Finally, the effect of incretins on insulin secretion, which is now highlighted in the context of treatment of diabetes and bariatric surgery, could be a powerful tool for decreasing postprandial glucose and for preventing diabetes. Long-term studies using incretin or dietary manipulations aimed at modulating their secretion are required.

#### Glucolipotoxicity of glucose on pancreas

To better understand the effect of hyperglycaemia on metabolic disease genesis, we can consider the potential toxicity of glucose, especially on the pancreas. It has been well documented that supraphysiological glucose concentrations may damage a large number of organs in non-controlled diabetes. However, chronic hyperglycaemia may promote the development of the early diabetic state by affecting the secretion capacity of pancreatic cells, which may subsequently increase blood glucose concentrations further. This vicious circle finally leads to the total failure of β-cells to secrete insulin (211). Moreover, excess of lipids added their deleterious effect to hyperglycaemia, leading to the concept of glucolipotoxicity. This could perhaps underlie the decrease in β-cell mass documented in patients with IGT, the earliest manifest stage of T2DM (212). These two recent reviews (211,212) synthesized the role of hyperglycaemia in the glucotoxicity. Long-term hyperglycaemia is able to induce the generation of ROS leading to chronic oxidative stress because the islets express very low levels of antioxidant enzymes and activity. However, these findings for chronic hyperglycaemia have not been demonstrated for postprandial glycaemic excursions.

#### Lipids and muscle IR

An increasing number of studies have linked lipid accumulation in skeletal muscles to reduced insulin sensitivity in T2DM patients. In addition, intracellular lipid metabolites, such as fatty acyl-CoA, diacylglycerol or ceramide, have been shown to inhibit insulin action via the activation of serine/threonine kinases and serine phosphorylation of insulin receptor substrate-1. There are a lot of experimental pieces of evidence showing a link between FFA level and IR. Lipid infusions, combined with heparin to activate lipoprotein lipase, raised plasma concentrations of fatty acids, promoted the accumulation of muscle lipids and impaired oral glucose tolerance and insulin-stimulated glucose disposal in healthy individuals (213). In addition, there is growing evidence that besides lipid overflow and an increased supply of fatty acids, mitochondrial dysfunction in skeletal muscles, and subsequently an impaired ability to oxidize fatty acids, also plays an important role in the development of IR. Bonnard *et al*. (214) have shown that both hyperglycaemia and hyperlipidaemia are involved in oxidative stress and mitochondrial alterations.

#### Liver metabolism: impact of insulin and glucose

The liver plays a major role in the regulation of glucose, lipid and energy metabolism. Acute elevations of circulating glucose concentrations decrease hepatic glucose output by acting on gluconeogenesis at the level of the expression of the PEPCK gene. The inhibitory effect of glucose on gluconeogenesis is affected during prolonged hyperglycaemia. However, the underlying mechanisms of this phenomenon have not yet been elucidated.

The brain has a substantial impact on the regulation of hepatic glucose metabolism. Moderate increases in extracellular glucose concentrations within a specific region of the hypothalamus markedly lower blood glucose through the inhibition of hepatic glucose production. Moreover, central inhibition of fat oxidation selectively activates specific neurons within the solitary tract nucleus and the vagal dorsal motor nucleus, leading to decreased levels of gluconeogenic enzymes and thus reduced hepatic glucose production. Hence, it has been shown that central glucose and lipid mechanisms communicate with the liver via the vagal nerve (215).

Under conditions of increasing glucose and insulin concentrations, lipid metabolism is affected by activating sterol response element-binding protein 1c (SREBP-1c) and carbohydrate response element-binding protein. Both are known to activate lipogenic enzymes in the liver (216). Recently, a long-term (20 weeks) study in mice showed that a high concentration of blood glucose through a high-GI diet induced the expression of enzymes involved in the regulation of *de novo* lipogenesis such as ACC and fatty-acid synthase, as well as stearoyl-CoA desaturase-1. In agreement with these findings, SREBP-1c was significantly increased (76). Furthermore, a network of transcription factors, repressors and activators act as sensors of hormonal and nutritional status (i.e. high glucose or insulin levels) in order to coordinate enzyme activities and metabolic pathways in the liver via Liver X receptors, which are recognized as important regulators of cholesterol metabolism, lipid and glucose homeostasis (216)).

#### Role of incretins

T2DM is characterized by a severely reduced or absent incretin effect (217) (see also the section on weight management in this article), and this undoubtedly contributes to the inappropriate insulin secretion that characterizes the disease. It is now clear that the incretin defect is due to an almost complete loss of the insulinotropic effect of GIP (218,219), whereas the secretion of GIP is normal or near normal (220). Regarding GLP-1, a significant and sometimes substantial reduction in meal-induced GLP-1 secretion is found (220,221). In contrast to GIP, the insulinotropic effect of GLP-1 is retained in patients with T2DM (218), and it is possible with infusions of GLP-1 to completely normalize glucose-induced insulin secretion in these patients (219,222). However, from dose-response studies it appears that the *potency* of GLP-1 with respect to enhancing glucose-induced insulin secretion is greatly reduced (to approximately 20%) in the patients compared with healthy controls (222). Thus, both defects in the secretion and actions of the incretin hormones are responsible for the reduced incretin effect and hence the inadequate insulin secretion seen in these patients. The question then arises whether these defects are primary or secondary in relation to diabetes, but the evidence to date suggests that the abnormalities are secondary to the development of diabetes and/or IR (223–226).

In contrast to GIP, with pharmacological amounts of GLP-1, it is possible to restore beta cell responsiveness to glucose in T2DM patients, and this is currently being exploited in the new therapeutic agents developed on the basis of GLP-1 (*exenatide*[92],[93],[95] and *liraglutide*[98]–[100],[102],[227]).

A dramatic example of the power of GLP-1 to regulate postprandial glycaemia is provided by diabetic patients undergoing gastric bypass operations for obesity. Up to 90% of these patients experience early remission of diabetes, independent of weight loss (whereas a similar weight loss alone can only explain a slow remission in up to 50%), and several studies have pointed to an exaggerated secretion of GLP-1 as an explanation for this (228,229). Interestingly, the human GLP-1 analogue, *liraglutide*, was given to a large group of non-diabetic overweight/obese subjects, of which one-third were glucose intolerant, for 20 weeks. During therapy (which led to significant and durable weight losses) glucose tolerance was normalized in 90% of participants (63).

### Conclusions

A large set of data going from the underlying mechanisms to long-term intervention studies support the role of a decrease in postprandial glycaemia in the prevention of diabetes. However, data are still lacking on which profile of the postprandial glycaemic response may be more favourable. More short-term interventions with careful control of these parameters are, therefore, still required.

Elevated blood glucose, concomitantly with elevated insulin concentration, leads to a transitory deleterious metabolic and hormonal state and oxidative stress, involving the liver, the pancreas, skeletal muscles, lipid metabolism interactions as well as incretins and inflammatory parameters in healthy subjects. These phenomena are exacerbated in participants with IGT. Some but not all studies have shown that reducing blood glucose and insulin responses is beneficial for preventing T2DM in at-risk subjects. However, based on the published literature, several clinical trials (short- and long-term) were excluded from the analysis because they failed to obtain significant differences in post-meal blood glucose responses. In fact, several studies using theoretically LGI vs. high-GI diets did not achieve differences in blood glucose response. One of the main issues is that the diet has to be well controlled and well selected to be able to provide a low glucose response via modifying the quality of carbohydrates, or by the addition of some soluble viscous fibres or other relevant diet/food manipulation. Thus, one of the challenges in clinical experiments, and moreover in ‘real life’, is to provide *really* low-glucose response diets. Then, there is a need for additional well-designed studies to confirm the metabolic impact of lowering blood glucose response concomitant with a low insulin response in healthy subjects.

## Cardiovascular prevention^4^

### Postprandial/post-challenge blood glucose and CVD in healthy people

The relationship between blood glucose levels and CVD in patients with T2DM has been well established by several epidemiological studies (230,231). However, the cardiovascular effects of intervention studies aiming at blood glucose optimization have not always given satisfactory results (232). Much of the epidemiological evidence (233,234) and some intervention studies in T2DM patients (235) support the role of postprandial blood glucose levels as an independent cardiovascular risk factor. The relationship between postprandial and fasting blood glucose, on the one hand, and cardiovascular risk, on the other, is more controversial in non-diabetic patients. First of all, it should be underlined that in relation to the postprandial phase, only data regarding post-challenge (i.e. 2 h after an oral glucose tolerance test [OGTT]) and not postprandial blood glucose levels are available, except for one study discussed later in this chapter. It is clear that these two metabolic conditions, although interrelated (236), are substantially different and, therefore, data based on post-challenge blood glucose should be extrapolated to post-meal blood glucose with caution.

The relationship between post-challenge blood glucose and cardiovascular risk in non-diabetic patients has been investigated in many prospective studies and reviewed in some meta-analyses (237,238). The largest meta-analysis by Levitan *et al*. (238) included 13 studies with post-challenge blood glucose data. This meta-analysis showed that

1. Both fasting and post-challenge blood glucose levels are significantly and independently related to cardiovascular risk in the non-diabetic glucose range.
2. The cardiovascular risk appears similar for fasting and post-challenge blood glucose levels, with 27% greater risk in individuals with the highest post-challenge blood glucose levels (150–194 mg dL^−1^) vs. those with the lowest ones (69–107 mg dL^−1^).
3. The relationship between post-challenge blood glucose levels and CVD seems to be continuous, at odds with that for fasting blood glucose, which seems to have a threshold at 100 mg dL^−1^. However, a minority of studies also show a threshold relationship for post-challenge blood glucose (239–245).
4. The relationship with both fasting and post-challenge blood glucose remains significant, although not equally strong, after adjusting for the main cardiovascular risk factors.

These data have been confirmed by the results of two more recent studies. One is a population study on a large number of men and women, which showed that both fasting and post-challenge blood glucose levels, even in a non-diabetic range, were significant and independent predictors not only of cardiovascular mortality but also of total mortality and that, also in this population, the type of relationship with post-challenge blood glucose levels was continuous (246). The other study showed that, even among individuals with both fasting and 2 h post-challenge blood glucose within the normoglycaemic range (i.e. individuals without impaired fasting glucose or IGT), those whose post-challenge blood glucose levels did not return to their fasting levels had a higher cardiovascular mortality than those whose post-challenge blood glucose returned to fasting levels (247).

The possible role of post-challenge glycaemia in determining a higher atherosclerotic risk has been investigated also in relation to surrogate markers of CVD, in particular to intima-media thickness (IMT). Studies indicate that post-challenge blood glucose levels in non-diabetic individuals are significantly associated with higher IMT independently of, and stronger than, fasting blood glucose levels (248,249). In one of these studies that evaluated different parameters during OGTT, 2 h post-challenge levels and 2 h post-challenge spikes (the difference between the maximal post-challenge glucose level and the fasting level) were significantly related to IMT; however, in the multivariate analysis only 2 h post-challenge levels remained significant (249). Unfortunately, a group of newly discovered individuals with T2DM were also included in this study, which prevented a clear conclusion about the role of post-challenge blood glucose levels in a non-diabetic range. All the principal prospective studies evaluating the relationship between post-challenge blood glucose and CVD in non-diabetic individuals are reported in Table 6. The only study performed measuring post-meal blood glucose has been carried out in participants with coronary artery disease. In this study postprandial blood glucose levels between 140 and 199 mg dL^−1^ were not associated with an increased cardiovascular risk (250). In conclusion, on the basis of the available epidemiological studies, there seems to be an independent association between post-challenge blood glucose and CVD in both non-diabetic men and women. However, it is not clear to which extent higher levels of this post-challenge as well as of fasting blood glucose levels in a non-diabetic range may help identify people at increased cardiovascular risk. In any case, because the relationship does seem to exist, it is important to (i) clarify the plausible underlying mechanisms of this association and (ii) to see whether any intervention studies are able to prove a causal relationship.

**Table 6:** Epidemiological studies evaluating the association between post-challenge blood glucose (2 h after 75 g glucose load) and cardiovascular disease in non-diabetic people

a. Studies comparing top and bottom categories of blood glucose						

Reference	Participants (*n*)	Follow-up (year)	Age (year)	Men (%)	Cardiovascular outcome	RR (CI)
Vaccaro *et al*. (1992) (441)	838	19	34–65	100	CVD mortality	1.96 (1.11 ± 3.45)
Fujishima *et al*. (1996) (240)	2,167	5	40–79	43	CVD mortality	1.9 (1.2 ± 3.2)
Park *et al*. (1996) (241)						
Men	549	8	55–90	100	CVD mortality	0.83 (0.47 ± 1.45)
Women	690	8	55–92	0	CVD mortality	1.01 (0.51 ± 2.00)
Lowe *et al*. (1997) (239)						
Black	594	22	35–64	100	CVD mortality	1.17 (0.66 ± 2.1)
White	9,830	22	35–64	100	CVD mortality	1.04 (0.91 ± 1.19)
Balkau *et al*. (1998) (242)	10,025	20	44–55	100	CVD mortality	1.08 (0.79 ± 1.50)
Rodriguez *et al*. (1999) (243)	6,514	23	48–68	100	CHD incidence	1.08 (1.11 ± 3.45)
Tominaga *et al*. (1999) (442)	2,398	6	≥40	44	CVD mortality	2.22 (1.08 ± 4.58)
DECODE Study Group (443) (2001)						
Men	14,376	8.8	30–89	100	CVD mortality	1.34 (1.12 ± 1.60)
Women	6,686	8.8	30–89	0	CVD mortality	1.28 (0.88 ± 1.86)
Sayadah *et al*. (2001) (244)	3,092	16	30–74	46	CVD mortality	0.93 (0.57 ± 1.51)
Smith *et al*. (2002) (245)	4,014	8.5	≥65	40	CVD incidence	1.90 (1.51 ± 2.39)
Nakagami (444) (2004)	6,817	10	30–89	45.7	CVD mortality	1.27 (0.86 ± 1.88)
Wild *et al*. (2005) (445)	1,496	15	55–74	50	CVD mortality	1.08 (0.34–3.40)
Pankow *et al*. (2007) (446)	6,888	6.3	52–75	42	CHD incidence	0.83 (0.59–1.17)
Wang *et al*. (2007) (447)	1,025	13	65–74	36.2	CVD mortality	1.55 (1.17–2.05)
Barr *et al*. (2007) (448)	10,428	5.2	58	40.7	CVD mortality	1.5 (1.1–2.05)
Chien *et al*. (2008) (449)	2,165	10.5	>35	44	CHD incidence	2.05 (1.23–3.42)
Succurro *et al*. (2009) *	400	–	21–70	44.7	IMT	*P* = 0.006
Cedeberg *et al*. (2010) (246)	593	10	61–73	41.3	CHD Incidence or CVD mortality	1.65 (1.19–2.30)
Ning *et al*. (2010) (247)						
Men	12,563	9	25–90	100	CVD mortality	1.22 (1.05–1.41)
Women	10,877	9	25–90	0	CVD mortality	1.40 (1.03–1.89)

b. Studies evaluating linear relationship of post-challenge blood glucose with CVD						

Reference	Participants (n)	Follow-up (y)	Age (y)	Men (%)	Cardiovascular Outcome	RR (CI)
Orencia *et al*. (1997)*(450)						
Men aged 18–39	10,269	22	18–39	100	CVD mortality	1.11 (0.97–4.26)
Men aged 40–59	7,993	22	40–59	100	CVD mortality	1.09 (1.04–1.14)
Men aged 60–74	1,240	22	60–74	100	CVD mortality	1.05 (0.96–1.14)
Women aged 40–59	6,319	22	40–59	0	CVD mortality	1.10 (1.00–1.21)
Women aged 60–74	932	22	60–74	0	CVD mortality	1.18 (1.06–1.31)
De Vegt *et al*. (1999) (451)	2,199	8	50–75	46	CVD mortality	2.24 (1.70–2.94)
Temelkova-Kruscev *et al*. (2000) †	582	–	40–70	47.7	IMT	1.50 (1.08–2.10)
Agewall (2001) (452)	113	6.3	50–72	100	CVD mortality	1.56 (1.03–2.48)
Meigs *et al*. (2002) (453)	3,370	4	26–82	46	CVD Incidence	1.18 (1.10–1.27)
Stern *et al*. (2002) (454)						
Men	1,693	7.5	25–64	100	CVD incidence	1.35 (1.16–1.57)
Women	2,209	7.5	25–64	0	CVD incidence	1.31 (1.11–1.54)
Barr *et al*. (2009) (455)	10,026	7	≥25	45.2	CVD mortality	1.2 (1.0–1.2)

* 1 h after 50 g glucose load.

† Postprandial glucose spikes (difference between the maximal PG level during OGTT, irrespective of the time after glucose challenge, and the level of FPG).

CHD, coronary artery disease; CI, confidence interval; CVD, cardiovascular disease; FPG, fasting plasma glucose; IMT, intima-media thickness; PG, postprandial glucose; RR, relative risk.

Different mechanisms may link non-diabetic hyperglycaemia with atherogenesis (251). Increased levels of non-diabetic post-challenge blood glucose generally identify people with IGT and/or other metabolic abnormalities such as overweight/obesity, insulin-resistance, higher levels of blood pressure, fasting and postprandial lipid abnormalities. Each of these metabolic abnormalities increases cardiovascular risk *per se*(252,253). However, the relationship between non-diabetic post-challenge hyperglycaemia and CVD is only partly explained by these other risk factors because it remains significant after also adjusting for these factors (238). Therefore, other mechanisms more strictly linked to hyperglycaemia *per se* should play a role, such as non-enzymatic glycosylation of proteins, increased glucose metabolism through the polyol and glucosamine pathways, and generation of free radicals, which could represent the unifying mechanism of hyperglycaemia-induced tissue damage (for more details on these points refer to other sections).

Intervention studies able to prove a cause–effect relationship are extremely rare. In the STOP-NIDDM trial in people with IGT, the administration of acarbose, which delays absorption of carbohydrates in the small intestine, resulting in more carbohydrates being subject to fermentation in the colon, resulted in a significant 49% RR reduction of overall cardiovascular events, which, however, were only secondary objectives of the study (3) (Table 7). The reduction in cardiovascular events was ascribed to a decrease in postprandial blood glucose levels, which were not measured in the study. However, the release of GLP-1 stimulated by acarbose may have also positively influenced cardiovascular risk. Very recently the NAVIGATOR study has shown that nateglinide, a drug able to reduce postprandial blood glucose, was unable to decrease cardiovascular events in a population at high risk of diabetes (197) (Table 7). As also indicated in the section ‘Prevention of diabetes and IR’ in this article, the difference between the two studies could be due to the different mechanisms of action of acarbose (decrease in postprandial blood glucose by delaying carbohydrate absorption) and nateglinide (decrease in postprandial blood glucose by enhancing first-phase insulin secretion) or, more simply, to the fact that in the NAVIGATOR study, nateglinide was actually not able to reduce postprandial blood glucose levels.

**Table 7:** Intervention studies evaluating the effect of postprandial blood glucose reduction on CVD

Study	Participants	Study design	Intervention	Follow-up (year)	Cardiovascular outcome	Effects
Chiasson *et al*. (2003) (3)	1,368 (*n*) with IGT	International multicentre randomized, double blind, trial	Acarbose x3 die vs. placebo	3.3	CHD incidence	↓
NAVIGATOR trial (2010) (197)	9,306 (*n*) with IGT	Randomized, double blind, trial; 2-by-2 factorrial design	Nateglinide + Valsartan vs. placebo	5	CVD mortality and incidence	=

CHD, coronary artery disease; CVD, cardiovascular disease; ↓, reduced incidence; =, no effect.

### Modulation of postprandial blood glucose response by different types of carbohydrates and its relationship with cardiovascular risk

As increased postprandial/post-challenge blood glucose levels are an independent cardiovascular risk factor, even in a non-diabetic range, it is important to modulate postprandial blood glucose in everyday life at the population level in order to reduce cardiovascular risk. It is well known that both the quantity and the quality of carbohydrates – best represented by their GI and/or GL – are the main modulators of postprandial blood glucose concentrations; it is, therefore, necessary to examine the data linking dietary GI/GL with (a) CVD or (b) the main cardiovascular risk factors.

- In the Nurses Health Study, Liu *et al*. (254) found a significant positive association between dietary GL and the risk of coronary artery disease after adjustment for known confounders, especially in women with a BMI > 30 kg m^−2^. This result has been confirmed by a recent study in women (255) but not by two other studies in men (256,257). However, in one of the latter studies a significant association was found in the group of men over 60 years with a BMI greater than 25 kg m^−2^. Therefore, a low GL, which can be due to a low carbohydrate content and/or to a higher intake of LGI foods, may be more beneficial in terms of cardiovascular risk in overweight people, who are at higher risk for diabetes and CVD. Data from a meta-analysis of observational studies confirm that diets with a high GI or GL independently increase the risk of heart disease (by 25%) and other chronic diseases (T2DM, breast cancer and gall bladder disease), showing that overall, the magnitude of the association was greater for GI than for GL (124). The data rom this meta-analysis are based on a population largely represented by women; therefore, these findings cannot be generalized to men. In this respect, results from a large cohort of Italian people confirm that a high dietary GL and, in particular, carbohydrates from high-GI foods increase the risk of CHD in women but not in men (258). Among the different types of carbohydrates, dietary fibre, one of the main contributors to the LGI and GL of a diet, is essential in the modulation of postprandial blood glucose and in the decrease of other main cardiovascular risk factors (such as LDL cholesterol, both fasting and postprandial plasma TGs, and blood pressure) (259–261). On the basis of these multiple beneficial effects, a significant inverse relationship has been found between the intake of dietary fibre, both soluble and insoluble, and CHD mortality (262–264). The studies evaluating the association between GI, GL, fibre intake and CVD in non-diabetic people are summarized in Table 8.
- With respect to the association between GI/GL and the main cardiovascular risk factors, epidemiological evidence shows
  1. An inverse relationship between the GL/GI of the diet and the level of HDL cholesterol in different countries and population groups (186,265,266).
  2. A positive association between the GL/GI of the diet and the concentration of fasting TGs, and this relationship is stronger in people with a higher BMI (186).
  3. A high GI or GL influenced the development of the metabolic syndrome in the Framingham Offspring Study (267)
  4. IR was associated with increasing GI in the Framingham Study but not in other studies (268,269).

**Table 8:** Epidemiological prospective studies evaluating the association between dietary glycaemic index, load and fibre intake with CVD

Study	Participants (*n*)	Follow-up (year)	Age (year)	Men (%)	Cardiovascular outcome	Risk in highest vs. lowest quantile		
						
						GL	GI	Fibre intake
Liu *et al*. (2000) (254)	75,521	10	38–63	0	CHD incidence	↑	↑	NE
Eshak *et al*. (2010) (263)	58,730	14	40–79		CVD mortality	NE	NE	↓
Van Dam *et al*. (2000) (456)	394	10	64–84	100	CHD incidence	NE	=	NE
Bazzano *et al*. (2003) (262)	9,776	19	25–74		CHD incidence	NE	NE	↓
Oh *et al*. (2005) (457)	78,779	18	30–55	0	Stroke incidence	↑ if BMI > 25	=	↓
					Ischaemic	=	↑ if BMI >25	=
					Haemorrhagic	=	=	↓
Beulens *et al*. (2007) (255)	15,714	9	49–70	0	CVD incidence	↑	↑	NE
Levitan *et al*. (2007) (256)	36,246	5	45–79	100	CVD mortality	=	=	NE
					CHD incidence	=	=	NE
					Ischaemic stroke incidence	=	=	NE
					Haemorrhagic stroke incidence	↑	=	NE
Wolk *et al*. (1999) (264)	68,782	10	37–64	0	CVD mortality or CHD incidence	NE	NE	↓
Mursu *et al*. (2009) (257)	1,981	16.1	42–60	100	CHD incidence	↑ if BMI > 25 and/or low physical activity	↑ if BMI > 25	NE
Levitan *et al*. (2010)*(458)	4,617	6	45–79	100	CVD mortality	=	=	NE
Hardy *et al*. (2010) (459)	13,051	17	45–64		CVD incidence	↑	↑	NE
					Ischaemic	=	=	NE
Grau K *et al*. (2010) (462)	2,790	6–25	30–70	50.7	CHD incidence			
Women						=	=	NE
Men						↓	=	NE

* In people with previous CVD.

BMI, body mass index; CHD, coronary artery disease; CVD, cardiovascular disease; GI, glycaemic index; GL, glycaemic load; NE, not evaluated; ↑, increased risk; ↓, reduced risk; =, no association.

Which mechanisms may explain the above associations? Diets with a high GI/GL and, in particular, with high GI and low fibre are associated with higher blood glucose levels, especially in the postprandial phase, and higher insulin demand, which leads to hyperinsulinaemia mainly in non-diabetic patients. Both higher blood glucose and insulin concentrations may lead to increased cardiovascular risk either directly or indirectly through the association with other cardiovascular risk factors such as hypertriglyceridaemia, low HDL cholesterol levels, hypertension and hypercoagulability. Thus, there is enough epidemiological evidence that diets with a high GL and/or a high GI may be detrimental in relation to CVD, and plausible mechanisms have been singled out.

However, epidemiological evidence must be supported by intervention studies. Unfortunately no intervention study has yet evaluated the effects of diets with different GL/GI and/or different amounts of dietary fibre on cardiovascular events. Many studies have evaluated, instead, the effects of these types of dietary intervention on postprandial blood glucose, insulin levels/IR, subclinical inflammation and oxidative stress (these issues are treated in other parts of the section). With respect to other cardiovascular risk factors, it is also clear that diets low in GL/GI and/or rich in dietary fibre are able to reduce LDL cholesterol, postprandial TG-rich lipoproteins and blood pressure in both diabetic and non-diabetic people. Moreover, in healthy subjects, LGI foods have been shown to produce a so called ‘second meal effect’ with improvement in post-lunch glucose, insulin and TG levels after a breakfast containing LGI foods compared with after a breakfast with high-GI foods (270).

### Conclusions

Epidemiological evidence indicates an independent relationship between post-challenge blood glucose levels and CVD in both diabetic and non-diabetic people, and this relationship seems to be continuous, i.e. without any real threshold. These data may be extrapolated with some caution to postprandial blood glucose levels for which, at the moment, there is no direct evidence.

The direct association between high-GI/GL diets, able to increase postprandial blood glucose, and CVD seems to support the role of postprandial blood glucose levels as an independent cardiovascular risk factor. It must be considered, however, that LGI diets, particularly if rich in dietary fibre, may also reduce cardiovascular risk through their action on other risk factors, especially LDL cholesterol and SCFA formation, during fermentation in the colon.

Finally, we need conclusive evidence, possibly from specific intervention studies with cardiovascular outcomes. In the meanwhile, our present knowledge is certainly enough to suggest LGI foods, particularly those rich in dietary fibre, as one of the components of a healthy dietary pattern for the prevention of CVD.

## Postprandial glycaemia and physical activity^5^

Depending on exercise intensity and duration, physical work may have profound effects on the utilization of glucose as a fuel in muscles. During low-grade intensity exercise, postprandial glucose levels are very similar to those during rest in both trained and untrained individuals who show similar metabolic responses following high-glycaemic or low-glycaemic foods. However, during intensive muscle contractions an enhanced glucose uptake from the blood will occur and thus the rate of ‘glucose disappearance’ from the circulation after a meal preceding exercise or, taken during exercise, will be enhanced. The effects of muscle contraction on glucose uptake from the blood are distinct from the effects of insulin and are known to continue even for many hours post-exercise. These effects of exercise lead to both a reduced post-exercise need for insulin production as well as to an enhancement of insulin sensitivity. Because of these observations, regular exercise is recommended as an important measure to manage postprandial blood glucose within ‘more healthy levels’ in patients suffering from T2DM. In the next paragraphs the effects of exercise on postprandial glucose regulation and utilization will be described.

### Role of carbohydrate stores and glucose availability during exercise

It is well established that maintaining a high glucose availability by consumption of carbohydrate during high-intensity exercise as well as ultra-endurance events can delay fatigue and thereby improve physical as well as mental performance (271–275). As intensive exercise induces hormonal, metabolic and circulatory changes that impact significantly on the fate of postprandial glucose and lipid metabolism, it is important to understand the differences from resting conditions. Carbohydrate, by means of glucose availability, is the most important fuel for high-intensity muscular work. During exercise, the muscle uses glucose taken up from the blood as well as that obtained from glycogen in muscle cells. In the body, glucose is stored as glycogen in the liver and in the muscle. The amount of glycogen stored in the liver amounts to approximately 100 g. This quantity may change periodically, depending on the amount of glycogen that is broken down for the supply of blood glucose, in periods of fasting and the amount of glucose that is supplied to the liver after food intake. Accordingly, liver glycogen reserves increase postprandially but diminish in between meals, especially during the night, when the liver constantly delivers glucose into the bloodstream to maintain a normal blood glucose level (276–280). During physical exercise, metabolic and hormonal stimuli as well as muscle contraction-induced stimuli will lead to an increased uptake of glucose from the blood. To avoid the blood glucose level falling below normal, the liver is stimulated to degrade glycogen for the delivery of glucose to the bloodstream. Thus, appropriate glucose storage in the liver is a key factor for the maintenance of a normal blood glucose level during prolonged exercise. Lack of liver glucose output may cause glucose to fall to hypoglycaemic levels and fatigue may develop. The amount of glucose that is stored in total muscle glycogen in the body amounts to approximately 300 g in sedentary people and may be increased to >500 g in trained individuals by a combination of exercise and the consumption of a carbohydrate-rich diet (276,281,282). The total intramuscular stored glucose, in energetic equivalent, may thus range from 1,200 to 2,000 kcal. The rate at which glucose is mobilized from muscle glycogen as a fuel for muscle contraction depends on a range of factors of which acute changes in energy requirements (exercise intensity) and availability of glucose and fatty acids for energy delivery are the most important. A very small pool of energy-rich phosphates (ATP and CP) is immediately available for muscle contractions at any moment of suddenly increased energy need, e.g. a sprint. These sources deliver most of the required energy for a period of up to maximally 10–15 s. For any longer-lasting events, the energy requirements will have to be covered almost completely by metabolism of glucose and fatty acids (276,279–281,283–285). The use of any of these two will never be exclusive. Thus, at any time muscle will use a mixture of carbohydrate and fat (and also, to a small degree, some protein). The most important factors that determine the relative contribution of carbohydrates, fat and aminoacids to the fuel mix and the rate and the extent to which macronutrient stores will be utilized are (i) exercise intensity and duration; (ii) training status and (iii) macronutrient ingestion.

#### Exercise intensity and duration

At rest most of the actual energy needs in muscle tissue will be derived from fat. In this situation the possible energy supply ratio may be in the order of 90% from fat and about 10% from carbohydrates. During a situation of increased physical activity, i.e. light physical work, or a moderately intensive sports activity, the body will use metabolic, hormonal and nervous control mechanisms to mobilize glucose from glycogen pools to serve as a rapidly available energy source (286). Synchronically an elevated mobilization of fatty acids will be initiated. After some time a new metabolic steady state will be achieved in which the energy supply ratio of fat to carbohydrates may then, for example, be about 50% : 50%. Thus, a gradual shift from high-fat/low-carbohydrate utilization at rest to enhanced carbohydrate/reduced fatty acid utilization during physical activity takes place. The absolute and relative contribution of carbohydrates and fatty acids to the fuel supply is influenced not only by exercise intensity but also by duration. Fat oxidation increases and carbohydrate oxidation decreases as the exercise duration increases. Typical fat oxidation rates are between 0.2 and 0.5 g min^−1^, but values of over 1.0–1.5 g min^−1^ have been reported after 6 h of running. The contribution of fat to energy expenditure can even increase to as much as 90% (287). With increasing work intensities, the body will start to use more and more carbohydrates as fuel. Accordingly, during all-out sports activity events lasting 1–3 min, carbohydrate will become the most important fuel (282,288,289). The reason for this shift to the dominant use of carbohydrate is that the amount of oxygen required for energy production from carbohydrates is about 10% lower than that of fat (290). Such a fine-tuned regulation of energy-rich substrate selection enables athletes to work at a higher intensity when using carbohydrates as the main energy source. Indeed, several lines of evidence show that intense and lasting muscle work cannot be performed without appropriate availability of carbohydrates and that athletes perform longer at high exercise intensity when glucose availability remains high (276,280,281,288,291). As such there is general consensus about the impact of glucose availability and the role of carbohydrate consumption during exercise. The authors of a very recent meta-analysis review concluded, based on 73 studies and 122 estimates, that the performance effects of carbohydrate supplements ranged from clear large improvements of ∼6% to clear moderate impairments of ∼2%. The best supplement inferred from the analysis consisted of a ∼3–10% carbohydrate plus protein drink providing ∼0.7 g kg^−1^ h^−1^ glucose polymers, ∼0.2 g kg^−1^ h^−1^ fructose and ∼0.2 g kg^−1^ h^−1^ protein (292).

#### Training status and gender

During high-intensity exercise, the time course of glycogen depletion will be influenced by the training status of the individual. Compared with less well-trained individuals, highly trained individuals have an improved capacity to mobilize fatty acids as a fuel. Thus, when working at a high but same absolute exercise intensity (e.g. running at a speed of 15 km h^−1^), trained individuals will use less carbohydrate and more fat for muscle contraction (284,293). At lower exercise intensities training status plays less of a role (294). Likewise, studies with trained and untrained sedentary individuals found similar metabolic responses following high-glycaemic or low-glycaemic foods and similar differences in substrate metabolism at rest and during low- to moderate-intensity exercise (295). Gender may impact, however, on substrate utilization in that greater fatty acid mobilization occurs in females during prolonged exercise compared with males. The reverse, however, is observed during the recovery phase, which may explain why, despite mobilizing lipids to a greater extent than males during exercise, females lose less fat mass than their male counterparts over the course of a physical training programme (296).

#### Postprandial glucose and type of carbohydrates consumed

The postprandial effects during exercise as well as the relative contribution of the individual substrates in the fuel mix used during physical activity depend on various factors, including the amount of carbohydrate ingested, the timing of intake and the intensity and duration of exercise (297). Generally, a high-postprandial blood glucose response following carbohydrate ingestion prior to exercise has large effects on insulin response, fat mobilization and substrate utilization during the onset of exercise. Lipolysis and fatty acid availability is suppressed, inducing a reduction in fat oxidation (287,298,299). Carbohydrate intake during exercise also inhibits fat oxidation by hindering the transport of fatty acids into the mitochondria (297). A lower postprandial blood glucose response as obtained following slowly digestible carbohydrate ingestion prior to and during exercise results in an accordingly lower rise in insulin levels and consequently in the maintenance of a higher rate of fat oxidation (298–301). Higher availability of fatty acids for oxidation, in turn, can cause a decrease in the rate of muscle glycogen breakdown (297). Further details on the metabolic effects of carbohydrate ingested during exercise and postprandial glycaemia, in particular on insulin release, absorption and oxidation during physical activity, are addressed below.

### Effect of postprandial glycaemia on substrate utilization and working capacity

#### Intake of carbohydrates reduces glycogen degradation

At low exercise intensities, the rate of utilization of glucose from stored glycogen in the body can be reduced by the consumption of carbohydrate. Any increase in blood glucose after oral carbohydrate intake reduces the need to break down liver glycogen for the maintenance of an appropriate blood glucose level. The reduction of liver glucose output equals the rate at which glucose is supplied to the blood from the intestine (302). The increase in blood glucose after carbohydrate intake during exercise will enhance glucose uptake by the muscle as well as subsequent carbohydrate oxidation. Several lines of evidence suggest that these events will help reduce the rate of muscle glycogen degradation (303–310).

As glycogen depletion and related falls in blood glucose during endurance exercise rarely take place when exercise duration is <30–45 min, guidelines for carbohydrate ingestion during exercise are primarily given for durations exceeding 45 min. Thus, for exercise lasting longer than 45 min it is recommended that at least 20 g, but optimally up to 60 g, be consumed, with sufficient fluid, for every next hour of exercise (288,311).

#### Carbohydrate types determine rate of glucose availability

The source and the molecular structure of carbohydrate will determine its rate of digestion, absorption and subsequent fate in metabolism. For example, foods that are rich in viscous dietary fibres delay gastric emptying and impact on intestinal viscosity, resulting in a reduced rate of digestion and absorption of glucose into the circulation. This would be a disadvantage in any condition requiring high glucose availability, such as during intensive physical endurance work. Several studies have addressed the effect of high- vs. low-glycaemic carbohydrate feeding on substrate and glycogen utilization. Generally it was observed that pre-exercise carbohydrate feeding with high-GI carbohydrates facilitates carbohydrate utilization during exercise (312,313). Studies have shown that different carbohydrates consumed during exercise are emptied from the stomach and are absorbed at different rates in the gut, while osmolarity does not play an important role at low carbohydrate concentrations as usually consumed during exercise (314–316). The intestinal absorption rate will strongly affect the glycaemic and insulinaemic responses and uptake in tissues, thus the availability for energy metabolism. For example, glucose is rapidly absorbed and oxidized compared with fructose and galactose, which are less readily absorbed (306,317,318). Incomplete absorption of carbohydrate ingested during exercise may lead to intestinal side effects due to carbohydrate accumulation in the intestine whenever the supply exceeds the rate at which the carbohydrate is absorbed. Some authors have reported intestinal upset when >30 g L^−1^ fructose has been ingested, both at rest and during exercise (319). However, in one study, this effect with ingestion of up to 1 g kg^−1^ body weight during exercise (320) was not found. Additionally, the rate of fructose utilization as a fuel during exercise has been shown to be lower. This may be caused by various events such as relatively slow fructose absorption, a significant metabolism of fructose during its first pass through the liver and by the fact that the enzyme hexokinase muscle has a much higher affinity for glucose than for fructose (303,304,311,318,319).

Factors that affect the rate of oxidation of ingested carbohydrates during exercise include feeding schedule, type and amount of carbohydrates ingested, and the exercise intensity. Glucose is a carbohydrate that is oxidized quickly as it needs no digestion and is rapidly absorbed. Also, and glucose polymers (MDX) are known to be oxidized at rates of 0.45–1.16 g min^−1^(321). Simultaneous ingestion of glucose and fructose may even result in oxidation rates of 1.5–2.4 g min^−1^, possibly reflecting different absorption routes resulting in a higher quantitative supply rate to blood (321–323). Accordingley, it has been shown that a combination of glucose and fructose can impact more favourably on physical work capacity compared with glucose alone (324).

Recently, some studies have been addressing the postprandial glycaemic and insulinaemic effect of the disaccharides ISO (gluc-fruc) and trehalose (TRE) (gluc-gluc). ISO and TRE are both disaccharides suggested to be slowly digested, resulting in a slow rate of absorption. In a study by Achten *et al*. (300), it was found that ingesting ISO every 15 min during a 2.5-h exercise trial of moderate intensity resulted in a lower plasma insulin response at 30 min compared with SUC, while the blood glucose response did not differ between ISO and SUC. Mean and peak oxidation rates of ingested ISO were significantly lower than that of SUC and resulted in a higher fat oxidation. In another study, TRE ingestion, compared with glucose, also resulted in lower insulin and blood glucose levels (325). In a follow-up study (301) TRE was compared with maltose (MAL) and a water placebo. TRE oxidation was lower than the MAL oxidation rate and total fat oxidation tended to be lower in MAL compared with TRE (*P* < 0.06). The reduced exogenous carbohydrate oxidation rate of TRE compared with MAL was assumed to be due to a reduced digestion rate in the small intestine, causing a slower availability for absorption. These data support the notion that the type of carbohydrate consumed impacts on substrate availability and oxidation rate.

##### Dietary starches

Some researchers have specifically studied the rates of postprandial glucose oxidation from various starch types. This is possible by using starch that is naturally enriched with Carbon 13 (corn starch) or by adding a C13 glucose tracer to starch. Both methods have their limitations. C13 starch studies are difficult to perform in US populations because their high consumption of cornstarch-containing products causes high levels of endogenous C13 enrichment of glycogen and also fat depots. In a European population this is less of a problem (326). Alternatively a C14 glucose label can be added (327). Adding a C13 or a C14 label to a starch in order to quantify the rate of oxidation of the label and assume that this equals the rate of starch oxidation is a practice that has been used. It has also been assumed that the rate of digestion of starch is non-limiting and that glucose derived from starch digestion behaves similarly to the glucose label added (327). However, Saris *et al*. (316) were able to show that this leads to an overestimation of the oxidation rate. Accordingly, appropriate measures of postprandial starch oxidation can only be obtained using intrinsically labelled starch. This was performed by Guezennec *et al*. (328), who compared the rates of oxidation of bread, potatoes, rice, spaghetti and glucose, given as isocaloric meals 1 h before exercise. Their study showed that the glucose and insulin responses were as follows in descending order: glucose > potatoes > bread > rice > spaghetti. The oxidation rate of spaghetti was lower than that of glucose. The postprandial oxidation rate of gelatinized amylopectin equalled that of glucose while a progressive decline in oxidation rate was observed from gelatinized amylose and crude amylopectin to crude amylose. These data confirm earlier published data from van Amelsvoort and Weststrate (329). From the hormonal and metabolic data described above, it appears that the type of carbohydrate will determine its kinetic properties for use during exercise. Thus, high exogenous carbohydrate oxidation rates and a fast utilization can be achieved with carbohydrate sources that are easily and fully digestible and absorbable, while lower postprandial blood glucose and insulin responses are related to lower oxidation rates. This is in line with recent observations that altering the GI of the carbohydrate within a meal significantly changes the postprandial hyperglycaemic and hyperinsulinaemic responses in women. An LGI pre-exercise meal resulted in a higher rate of fat oxidation during exercise than did an HGI meal (295).

#### Postprandial macronutrient interactions influence the metabolic fate of glucose

The metabolism of carbohydrate may also be influenced by the presence of other macronutrients. For example, recent studies have shown that a mixture of 0.8 g × kg × h carbohydrate and protein or selected amino acids leads to a significantly higher insulin response compared with the ingestion of carbohydrate alone (330,331). This was assumed to be of advantage in circumstances where a high insulin level may be of benefit to the rate of uptake of glucose in tissues. Accordingly it was observed that the resynthesis rate of muscle glycogen after an exhausting exercise proceeds more rapidly when such a combination is ingested (331). However, at higher levels of carbohydrate intake, i.e. 1.2 g × kg^−1^ × h^−1^, the stimulating effect of insulin on glycogen recovery was not apparent anymore (332,333).

However, at higher levels of carbohydrate intake, i.e. 1.2 g × kg^−1^ × h^−1^, the stimulating effect of insulin on glycogen recovery was not apparent anymore (332). Accordingly, Cermak *et al*. (334) observed that when trained men ingest carbohydrate at a rate at the upper end of the range generally recommended to improve endurance performance, co-ingestion of protein does not alter specific markers proposed to reflect an enhanced capacity for skeletal muscle energy delivery.

#### Postprandial glucose homeostasis during exercise

During physical activity the muscle does not solely rely on insulin for the uptake of glucose (335–337). Muscle contractions mobilize glucose transporters (GLUT 4) in a similar way to insulin, but by a mechanism that is not yet fully understood. It is likely to involve Ca(2+)-calmodulin-dependent protein kinase, 5′-AMP-activated protein kinase and possibly protein kinase (338). During intense physical exercise this effect is explicitely required because muscle activity causes an intensity-dependent inhibition of insulin secretion through the action of the elevated catecholamines. This contraction-induced effect is referred to as ‘insulin-like action’, which may persist even for >12 h after exercise has been stopped. Accordingly, a high intake of carbohydrates during exercise may cause only a small elevation of insulin in the case of high intensity or a strong increase when exercise intensity is low. In the case that carbohydrate is consumed shortly before exercise, the combination of elevated insulin-induced glucose uptake + muscle-induced glucose uptake action may cause reactive hypoglycaemia because of muscle glucose uptake exceeding liver glucose output. Some early studies showed that an intake of 50–75 g of rapidly absorbable carbohydrate in a fasted state and shortly prior to exercise induced a rapid rise in blood glucose and insulin followed by a rebound hypoglycaemia as well as decreased performance during the subsequent exercise. However, these conditions may be different from those of an endurance athlete, who is advised to eat a pre-game breakfast and perform a warm-up prior to the start of the competition. Studies done under such real competition conditions with regular intake of carbohydrate during exercise did not show any rebound hypoglycaemia (339,340). In this respect, carbohydrate intake during exercise will also counter affect pre-exercise diet effects (341).

The occurrence of rebound hypoglycaemia may depend on the timing of carbohydrate intake. In a recent study the metabolic and performance responses to the ingestion of 75 g of glucose dissolved in 500 mL of water at differing times before exercise were investigated (342). Altering the timing of the ingestion of carbohydrate before exercise resulted in differences in plasma glucose/insulin responses, which disappeared within 10 min of exercise and which had no effect on performance. Postprandial rebound hypoglycaemia is independent of insulin sensitivity and exercise intensity and does not occur in all individuals, i.e. some individuals are more sensitive than others. Moreover, if present, this condition is not always associated with reduced performance capacity (342,343).

### Immediate post-exercise carbohydrate intake enhances physical recovery

Glycogen resynthesis has been shown to be most rapid during the first few hours after exercise when muscle glycogen is low, but only when carbohydrate has been consumed to supply the required glucose (276,288,304,311). The latter is influenced by the type of carbohydrate and its rate of digestion and absorption. No difference appears between males and females in their capacity to replenish glycogen stores; optimal timing for carbohydrate intake does not differ between genders, and athletes must consume carbohydrates as soon as possible after exercise in order to maximize glycogen store repletion (344).

Glucose favours muscle glycogen recovery, but fructose is primarily taken up by the liver and favours liver glycogen recovery (277,345,346). When the next activity takes place, after 1 or 2 d, the athlete can recover properly by ingesting normal meals with a high carbohydrate content, i.e. 55–65 en% (en% = percentage of total daily EI). These meals can best be composed of LGI foods such as whole grains, cereals, pulses, fibre-rich fruits and vegetables. A relatively slow digestion and absorption rate is favourable in this condition. Sports practice and also controlled experiments have shown that the high needs for energy and carbohydrate during days with an energy expenditure exceeding 4,500 kcal d^−1^ can only be covered appropriately by the ingestion of rapidly digestible foods/beverages that are high in carbohydrates (347–349). Carbohydrate solutions can be taken during exercise in any situation in which carbohydrate intake through the consumption of normal food cannot take place or is insufficient. This will help to enhance glycogen recovery in the first few hours after exercise (304,311,350). Extensive reviews can be found in the literature (271,292,296,344,351).

## Postprandial carbohydrate metabolism and cognitive performance^6^

### Introduction

Diet-related disorders such as obesity (352) and T2DM (353) are increasingly associated with cognitive decline (463). The emerging knowledge in this area has generated interest in the potential to prevent impairment of cognitive functions via choice of diet. Very little information is available concerning the long-term impact of different diets, and to our knowledge no information is available concerning the effects of dietary carbohydrate characteristics such as the GI in this respect. However, recent reports suggest that breakfast habits in children may influence brain grey-matter volume and that children with a higher habitual intake of rice (presumably LGI) display larger global grey-matter volume than those with a higher habitual intake of white bread (354).

With respect to acute studies regarding the effects of macronutrients on cognitive measures, carbohydrates were the most frequently studied and had the greatest positive effects on verbal and delayed memory (355). Glucose forms the major energy substrate for the brain and is fundamental for normal functioning of the central nervous system. The provision of glucose generally improves various measures of memory function compared with placebo (356–358). This facilitating effect is seen following the administration of carbohydrates after overnight fasting and in between-meal fasting situations (359). However, hyperglycaemia, especially oscillatory excursions as may occur in the postprandial phase in the diabetic state, may trigger oxidative stress and inflammation (360); see also the section on the prevention of diabetes and IR in this article, suggesting a potentially detrimental effect of high-GI foods.

Whereas high-GI foods are characterized by elevated glycaemic responses of short duration, LGI foods typically induce a lower peak response, and a more even and sustained provision of glucose to the blood in the postprandial period, hence counteracting hypoglycaemia which may occur with high-GI foods in the later postprandial phase (129,361). While the induction of hypoglycaemia in healthy subjects results in significant impairment of cognition (362), less is known about the influence of glycaemic excursions below fasting levels following the ingestion of high-GI meals. Interestingly, changes in metabolite concentrations were suggested to be more important determinants of cognitive function in the postprandial phase than their absolute values (363,364), indicating benefits of a balanced glucose metabolism and avoidance of oscillatory glycaemia. The present summary discusses available literature concerning the potential impact of the course of postprandial glycaemia on measures of cognitive function (Table 9).

**Table 9:** Available studies on the potential impact of postprandial glycaemia on cognitive function

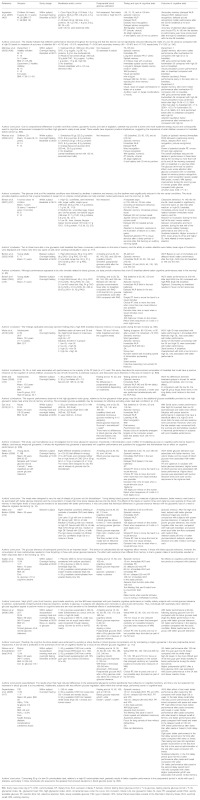

### Glucose and the brain

During cognitive effort, local extracellular concentrations of glucose decline in the brain areas activated. Visual stimulation can thus result in significant decreases in glucose concentration in the visual cortex in humans (365), and memory activation in rats causes reductions in hippocampal glucose (366). An equilibrium exists between plasma glucose concentrations and glucose in the extracellular fluid such that the concentration of glucose in the brain is 20–30% of that in blood plasma (367–369). Therefore, increasing plasma blood glucose levels results in a relative increase in brain extracellular glucose levels (370,371). This may enhance neuronal activity when brain extracellular glucose levels are depleted, which can occur as a result of high neuronal activity (372) or because of abnormalities in the transfer of glucose across the blood–brain barrier (BBB). The BBB breaks down with age (373–375). Therefore, when glucose in the extracellular fluid is reduced to a level that results in neurons performing at a suboptimum level, glucose consumption can facilitate neuronal activation. This mechanism could account for the observation that glucose consumption aids cognition during periods of high cognitive demand, and cognition is impaired in those with IGT (376,377).

The synthesis of several key neurotransmitters in the brain such as glutamate, gamma-aminobutyric acid and acetylcholine relies on a supply of glucose. Acetylcholine (from acetyl-CoA derived from glucose metabolism) and choline increases synaptic plasticity, important for learning and short-term memory. Also, glucose metabolism can affect the utilization of tryptophan and may increase serotonin concentration in the brain, hence affecting memory and mood.

### Cognitive functions in relation to glucose regulation and IR

Insulin is absorbed in the brain by insulin receptors (378–380) and areas of the brain, associated with learning and memory express a high density of these receptors (367). Therefore, a reduction of insulin in the brain or the presence of IR may cause neurons to be functionally insulin deficient (381). Increases in hippocampal insulin receptor expression and signalling are associated with improved spatial memory performance (382), and impairments in rodent hippocampal synaptic plasticity can be reversed by insulin replacement (383). In humans, intravenous or intranasal injections of insulin have been associated with cognitive improvements in healthy adults (384,385) and Alzheimer's patients (386–388). Furthermore, experimental studies have shown that drugs that improve insulin sensitivity (thiazolidinediones) are associated with cognitive improvements (389).

The glucose transporters GLUT 4 and GLUT 8, found throughout the brain, are also sensitive to insulin levels (390,391). This suggests that insulin also affects the transport of glucose within the brain. Chronic peripheral hyperinsulinaemia, as occurs in IR, negatively influences neuronal function and survival. Eventually, peripheral hyperinsulinaemia results in a reduction of insulin transport across the BBB and in reduced insulin receptor signalling, leading to reduced synaptic plasticity in the brain with effects on learning and memory (392). IGT with concomitant IR results in impaired cognitive functions (393–395). However, reduced glucose tolerance within the normal range can also be accompanied by impaired cognitive performance (377,396–399). Oxidative stress plays an important role in the pathogenesis of IR and beta-cell dysfunction. As oscillating hyperglycaemic episodes promote oxidative stress (360), it can be suggested that elevated postprandial glucose excursions could be detrimental for IR signalling in the periphery, with potential impact also centrally, and hence on cognitive function. Of interest, in connection with this, are data showing that venous administration of insulin increases insulin levels acutely in the cerebrospinal fluid (385). Also, exogenous administration of insulin under euglycaemic conditions in healthy subjects and subjects with Alzheimer's disease acutely improved measures of cognitive function (400). Hence, a habitual diet that maintains insulin sensitivity and IR signalling, and secures optimal glucose delivery to the brain in the fed and fasting states should be most advantageous for the maintenance of cognitive function. The glycaemic potency of the diet is of interest in this context. Studies are required to examine IGT and cognitive function in the absence of other comorbidities to determine the possible causal effect of IGT on cognitive function.

### Postprandial glucose metabolism in relation to cognitive function

The effects of breakfast on the cognitive performance of children and adolescents have recently been reviewed (355). Young children might be expected to be more susceptible to effects of brief or overnight fasting because of greater metabolic demands of the brain relative to liver and muscle glycogen stores, and childrens' lower capacity for gluconeogenesis (401–403). Wesnes *et al*. (401) compared the effects of consuming cereal breakfasts (Cheerios or Schreddies), glucose or no breakfast on cognitive performance in children (9–16 years). No glycaemic measurements were performed, and the breakfast meals consumed reflected realistic portion sizes. Consequently, about 38 g of available carbohydrates were provided by the Shreddies and the glucose drink vs. about 29 g with Cheerios. According to published GI (404), the GI of Cheerios and Shreddies are similar (about 84 and 75, respectively). The cereal products were ingested with milk, which is insulinotropic and would probably result in lower GIs than calculated from the individual carbohydrate components, as well as shortened blood glucose response profiles. Repeated batteries of computerized tests of attention, working memory and episodic memory were administered during the postprandial period (up to 210 min). The cereal breakfasts conferred benefits for cognitive functions compared with glucose or no breakfast, particularly towards the end of the morning, with little difference in between the two cereal breakfasts.

A study in younger children (6–11 years) consuming All bran (GI = 42) and Coco Pops (GI = 77) allowed a stronger comparison of the impact of varying breakfast GI (405) using the same tests as employed by Wesnes *et al*. (401) performed up to 130 min post-ingestion. The decline in performance of attention tasks in the postprandial period was more pronounced after the high-GI breakfast meal. The reduction in decline in children's performance throughout the morning was attributed to the LGI cereal breakfast. However, the ‘cognitively superior’ LGI breakfast, in addition to being ‘lente’, also provided less available carbohydrates, 16 vs. 30 g. In support of above, Mahoney *et al*. (406) evaluated cognitive outcomes after LGI (oatmeal) vs. HGI (ready-to-eat cereal [RTEC]), or no breakfast in two groups of children (6–8 years and 9–11 years, respectively). Breakfast cereals were served with skimmed milk and matched on total carbohydrates. Breakfast intake *per se* improved performance on tasks requiring the processing of complex visual display in the 9- to 11-year-olds compared with no breakfast. Better spatial memory and auditory attention following the LGI oatmeal breakfast compared with the HGI RTEC was shown in the younger children. All effects observed were in the late postprandial phase. Benton *et al*. (407) provided three composite breakfast meals varying in both GI and GL to school children (5–7 years). The consumption of a low-GL meal was associated with better scores on memory, ability to sustain attention and improved time spent on specific tasks in the classroom. Differences in GL were achieved by varying the GI of the breakfast products (GI 50–70) and the carbohydrate content of the breakfasts, with dairy products included in the high- and intermediate-GL breakfasts only, potentially lowering the range of GI. Benefits were observed on classroom behaviour, memory, reaction to frustration and sustained attention in the later postprandial phase (2–3 h after breakfast) with low GL (401,405). In a recent study in healthy adolescents (11–14 years) (408), cognitive performance was assessed and related to the GI and GL characteristics of breakfast estimated from self-reported data. Adolescents were divided into four groups depending on the character of the breakfast; low GL + LGI (*n* = 19), low GL + high GI (*n* = 11), high GL + LGI (*n* = 11) and high GL + high GI (*n* = 19), and cognitive functions were assessed between 90 and 120 min postprandially. Different aspects of cognitive performance were associated with each breakfast, e.g. high GL + LGI produced better information processing and working memory performance in tasks that the subjects found ‘most difficult’, while high GI and high GL breakfasts were associated with better immediate word recall and matrices performance, respectively.

Studies have also been performed in young healthy adults. Benton and co-workers (409) investigated the impact of two cereal breakfasts in young females. The breakfast meals contained similar carbohydrate contents and GI characteristics were predicted from *in vitro* enzyme availability data (42 and 66, respectively). Glycaemic response was measured for 230 min after breakfast while episodic memory was recorded up to 220 min. The LGI breakfast significantly improved memory function, particularly in the late postprandial phase (150 and 210 min after breakfast), with no concomitant differences in blood glucose. Hence, cognitive outcomes could not be attributed to late glycaemia *per se*. Other studies have, however, failed to show significant differences in cognitive functions related to the GI of breakfast meals in this age group. In a study in adolescents (14–17 years) (410), an LGI (All bran GI = 30) or a high-GI breakfast (Cornflakes GI = 77) was provided after an overnight fasting. Glycaemic measurements were performed up to 90 min, but no significant differences in glycaemia were observed. Analysis of remembering/forgetting indices revealed that the high-GI breakfast induced cognitive benefits in comparison with the LGI breakfast. However, immediate free recall, and short and long delayed recall, respectively, tended to benefit from the LGI breakfast, although this effect may be due to the the way in which these scores were derived from subtests.

The rate of glucose delivery to the blood can be manipulated by altering the chemical or physical structure of the carbohydrate moiety, thus making it possible to examine the impact of inducing very different glycaemic responses on cognitive function (411). ISO, an isomer of SUC containing an α-1,6 glycosidic linkage, is considered slowly but completely digested and absorbed in the gastrointestinal channel, providing a low but sustained glycaemic response (412). ISO or SUC was consumed in milk-based drinks in the morning, and glycaemic profiles were measured continuously with cognitive performance assessed in the postprandial phase up to 165 min. Immediate and delayed verbal memory, psychomotor performance and serial sevens were assessed. Despite large and significant differences in the amplitude of postprandial glycaemia, no major differences occurred across the measures of cognitive function. Postprandial glucose and hormonal responses to drinks with ISO and SUC in healthy subjects have been reported in other studies using capillary blood (412). Interestingly, these studies have indicated not only a reduced initial glycaemic response following iso-maltulose but also a prolonged net increment in blood glucose in the late postprandial phase but failed to examine cognitive function. In contrast, in the only carefully controlled cognitive study of ISO to date, Dye *et al*. (411) demonstrated that capillary and interstitial blood glucose after both iso-maltulose and SUC had returned to, or below fasting level, respectively, after approximately 95 min, which is not consistent with a slow and more distal digestion and absorption. This could reflect the good regulatory capacity of the subjects or the effects of the milk-based vehicle where dairy proteins will dampen glycaemia but nevertheless demonstrates that even when the rate of glucose uptake differs significantly, cognitive effects are not necessarily observed.

Using a factorial design, cognitive functions were investigated in young female adults (*n* = 168) after eight breakfasts differing in GI (range 47–71), as well as in the amount of carbohydrates and dietary fibre (413). In subjects with poorer glucose tolerance, consuming more carbohydrates resulted in more forgetting. In contrast, higher amounts of carbohydrates improved reaction time after 90 min in the poor regulators. The lowest levels of fibre (1.5 g) were associated with poorer memory in subjects with poorer glucose tolerance. However, blood glucose responses were not affected by dietary fibre content, indicating that the expected variation in GI was not achieved in the composite meals but the timing of blood glucose sampling was such that differences may have been missed.

No differences in cognitive performance were found after test meals differing in GI in elderly subjects (60–82 years) (414). Three test meals provided 50 g of available carbohydrates from pearl barley (GI = 28), potatoes (GI = 56–118) (415), glucose (GI = 100), and a placebo drink with no carbohydrates and were consumed in a within-subjects design. Cognitive tests were administered 15 min after commencing the breakfast and 105 min postprandially. Performance after the consumption of potatoes or barley did not differ significantly from performance after the placebo on any cognitive tests. However, the potential impact of GI may have been obscured by the fact that not all subjects managed to consume the breakfast meal prior to cognitive testing. Nilsson *et al*. (399) tested working memory and selective attention in healthy normal-weighted middle-aged subjects after 50 g of glucose in drinks administered either as a bolus load, or by sipping to achieve a rapid high peak followed by a steep decrease to below the fasting value, or a smooth course of glycaemia, respectively. Better performance in working memory (90 min) and selective attention (170 min) in the later postprandial period was observed after sipping. In addition, subjects with relatively better glucose tolerance performed better on the cognitive tests than those with lower glucose tolerance (albeit in the normal range). The cognitive functions tested were enhanced by preventing a sharp decline in blood glucose concentration and by maintaining a higher glycaemia in the late postprandial period, respectively. The same authors (416) recently confirmed the cognitive benefits of a low but sustained net increment in blood glucose in the late postprandial phase using an LGI food prototype product compared with a high-GI breakfast matched on carbohydrate content and gross nutrient composition.

Similar effects on cognitive performance after breakfast have also been reported in type 2 diabetic subjects. Papanikolau and co-workers provided breakfasts consisting of pasta, toasted white bread or water (417). Higher postprandial blood glucose AUC was associated with poorer verbal memory, and in general the LGI pasta meal resulted in better performance. However, both bread and pasta resulted in higher scores for sustained attention than water. Interestingly, the superior performance with pasta was not seen in the earliest tests (15 min, immediate recall) but was apparent between 62 and 100 min in the postprandial phase.

### General discussion

Of the 15 studies shown in Table 6, 4 do not permit conclusions to be drawn regarding the possible relationship between postprandial glycaemia and cognitive performance (408,409,413,418). Of the remaining 11, 2 found no effects of GI characteristics (411,414), but while the design of the test breakfasts in these studies was appropriate to evaluate the potential influence of the GI, compliance to the breakfast meals in the Kaplan study was poor. One study reported benefits of HGI vs. LGI breakfasts (410) with the effect in question based on a calculated cognitive parameter.

In total nine studies showed cognitive benefits following LGI breakfasts. The quality of the evidence in these studies varies, and in many cases the GI of the test breakfasts are estimates from international tables, with no actual glycaemic profiles provided. Furthermore, in many experiments the test foods differ in a number of ways, not just in GI. The course of glycaemia following breakfast in relation to cognitive functions has been reported in six studies (399,409,411,416,417,419). With one exception (411), the findings favour LGI.

Of the four studies performed in children, three employed experimental designs that allow conclusions regarding the GI *per se*, or the GI character of the breakfast tested at realistic portion sizes (401,405,406). All three studies suggest benefits of LGI breakfast meals on the cognitive domains of memory and attention.

The impact of the course of glycaemia on cognition based on studies in young adults is inconsistent. The nature of the cognitive task may affect its susceptibility to enhancement by glucose (356,357,420), and in young insulin-sensitive subjects, effects on cognitive functions may be undetectable even if demanding cognitive tasks are employed.

Most studies report effects on cognitive outcomes in the later postprandial phase. Although benefits in cognitive function in the late postprandial phase coincided with higher late glycaemia in two of the studies (Nilsson *et al*. unpublished) (399), such benefits are also reported with LGI breakfasts in the absence of differences in blood glucose levels (409), suggesting that the provision of glucose from the circulation *per se* is not the only important factor for brain function. Instead, the improvement in cognitive functions seen in the late postprandial phase in some studies with LGI breakfasts may also be secondary to improved insulin sensitivity. Progressively, deterioration of glucose regulation predicted poorer performance on working memory and executive function in a group of healthy middle-aged to elderly subjects (397), suggesting that dietary interventions that improve glycaemic control are important to protect cognitive functions. In light of the role of insulin receptor activity in brain signalling (392,421–423), effects on postprandial insulin sensitivity may be a mechanism whereby LGI meals may confer benefits for cognitive performance (424). Improvements in glucose tolerance following an LGI meal can carry over from one meal to another, ingested 4 h later (113,270,425,426). Improved glycaemic regulation and lowered insulin demand at the lunch meal following LGI breakfasts has been attributed to the slow and sustained carbohydrate absorption resulting in lowered plasma FFA at lunch time (427). This hypothesis is substantiated by the fact that sipping glucose over 3.5 h, compared with a bolus load, resulted in a prolonged suppression of plasma glucagon, growth hormone and FFA in the late postprandial period (428). As FFA level is considered a marker of IR, this suggests that slow release carbohydrates may beneficially modulate insulin sensitivity in the postprandial phase, thus increasing glucose tolerance at a subsequent meal ingested 4 h later (464).

Taken together, there is plenty of evidence to support IR as a postprandial phenomenon, and that differences in GI and/or the course of postprandial glycaemia may be of importance in this context. The possible acute effects of a meal on insulin sensitivity and insulin receptor signalling is of potential relevance in relation to cognitive function because, as described above, suppression of insulin receptor signalling can compromise neuronal plasticity.

### Conclusions

The conclusions from studies available to date are tempered by a range of methodological limitations, e.g. poor descriptions of meals or products ingested as well as of cognitive tests administered, insufficient standardization of the available carbohydrate content and nutrient composition of the meals, lack of adequate information on, or physiological confirmation of, the course of postprandial glycaemia, insufficient duration of the meal test and subsequent test period, or too few test subjects. Taken together, the studies generally favour LGI meals for improved memory and/or attention, particularly in children and elderly, and mainly in the late postprandial phase. The beneficial effects may be secondary to a smoother overall blood glucose profile with sustained availability of glucose to the brain and/or to an acute improvement in insulin sensitivity. More studies are needed to deduce the impact of the course of glycaemia and related metabolic parameters such as inflammatory markers, and acute measures of insulin sensitivity on cognitive performance. Imaging techniques may provide valuable information in this context.

Studies are needed to elucidate the relationship between peripheral IR, as may occur in the postprandial phase, and insulin receptor activity in the brain. Also needed are studies of the impact of habitual consumption of low- vs. high-GI diets on IRS and cognitive performance.

## Overall conclusion

Tight glycaemic control is necessary to maintain health and to prevent disease. This review provides an overview of postprandial glucose in relation to body weight control, oxidative stress, T2DM and CVD. It also addresses the impact of exercise performance on blood glucose control and utilization and the relationship between blood glucose and cognitive function. Blood glucose responses are interrelated with insulinaemic and lipidaemic responses, and the mechanisms underlying blood glucose responses are related to food intake, insulin secretion capacity and insulin sensitivity. Thus, the blood glucose responses *per se*, as well as the interrelated factors and underlying mechanisms, have been discussed.

### Body weight control

Studies suggest that the types of foods that elicit a lower postprandial glucose response may be useful as part of an overall strategy for combating obesity and its associated burden of chronic disease, but the evidence for a direct role of postprandial glucose *per se* in these effects is considerably weaker. Overall, evidence from short-term studies of GI is more convincing than that from longer-term studies in terms of an effect on appetite regulation. However, it is not clear that effects attributed to GI are actually driven by variation in postprandial glucose responses *per se*, as studies directly testing this are unsupportive. There is also some evidence that LGI diets (via a slower uptake of carbohydrate) stimulate fat oxidation at the expense of carbohydrate oxidation in the short-term (e.g. Brand-Miller *et al*. (8)) and reduce body fat deposition (8,128). The mechanisms relating glycaemic response to the regulation of body weight have been examined in controlled feeding studies (129) and involve acute hormonal and metabolic changes that may decrease hunger and EI and affect nutrient partitioning and fat oxidation (8). Of particular interest are also gastrointestinal peptides that are released following food consumption and which are involved in the control of appetite and satiety (CCK, GLP-1, PYY and ghrelin).

There is currently little data on the chronic effects of reducing postprandial glycaemia on body weight. The recently completed Diogenes study has attempted to reduce this knowledge gap, showing clear positive effects of an LGI diet in reducing weight regain after weight loss. Again, it is not clear whether the effect of an LGI diet is driven by a reduced postprandial glycaemia *per se*, and multiple mechanisms may be responsible for the positive effect on body weight control. Recently published opinions on DRVs for carbohydrates, dietary fibre, fats and water, adopted by EFSA's Panel on Dietetic Products, Nutrition and Allergies (130), include the conclusion that evidence is still inconclusive on the role of GI and GL in maintaining weight. It is evident that there is a clear need for more long-term well-designed human studies to evaluate how variation in glycaemic response, induced by well-characterized low- or high-glycaemic diets, affect EI, body composition and body weight for biologically significant periods of time.

### T2DM

Prospective cohort studies comparing diets with different GI or GL have shown conflicting results with respect to developing T2DM (i.e. EFSA opinions [130]). The Barclay meta-analysis (187) included eight studies and reported that an increased risk of diabetes was found when comparing highest to lowest quintiles of both GI and GL. The meta-analysis of Livesey included experimental studies and showed that diets with an LGI have a beneficial impact on fasting glucose, glycated protein and insulin sensitivity in non-diabetic subjects. Unfortunately, insufficient data on postprandial glucose profiles were available.

There is mechanistic evidence from animal and human studies that elevated blood glucose and an altered pattern of blood glucose response (i.e. late hypoglycaemia), together with an elevated insulin concentration, leads to a transitory deleterious metabolic and hormonal state, which involves the liver, pancreas, skeletal muscle and lipid metabolism interactions as well as incretins and inflammatory parameters in healthy subjects. These phenomena are exacerbated in impaired glucose-tolerant subjects. Thus, there is proven evidence that reducing blood glucose and insulin responses is beneficial to prevent T2DM genesis. However, a problem in interpreting available literature is that several studies utilizing theoretically LGI vs. high-GI manipulations did not produce differences in blood glucose response. A selection of intervention studies with reported postprandial glycaemic profiles (*n* = 5) indicated that differences in postprandial blood glucose response should be more than 1.3 mmol L^−1^ and that insulin responses should be significantly different to obtain clear metabolic effects.

One of the main issues in these studies is that the diet has to be well controlled and well selected to be able to provide a low glucose response via modifying the quality of carbohydrates. Thus, one of the challenges in clinical experiments and moreover in ‘real life’ is to provide effective low glucose-response diets in order to draw a more definite conclusion on the role of postprandial glycaemia in the prevention of diabetes.

### Oxidative stress and CVD

Epidemiological evidence seems to indicate that there is an independent relationship between post-challenge blood glucose levels and CVD, even in non-diabetic people, and this relationship seems to be continuous without any real threshold. These data may be extrapolated with some caution to postprandial blood glucose levels for which, at the moment, we do not have direct evidence. The direct association between high-GI/GL diets, which are able to increase postprandial blood glucose, and CVD seems to support the role of the postprandial blood glucose level as an independent cardiovascular risk factor. In any case, it is important to consider that LGI diets, particularly if rich in dietary fibre, may reduce cardiovascular risk through their action on other risk factors, especially LDL cholesterol.

Whether postprandial glucose is a stronger risk marker for CVD as compared with fasting glucose or HbA1c remains controversial. Many studies have reported a stronger association of CVD risk and postprandial or post-load glucose than for CVD risk and HbA1c or mean blood glucose. However, HbA1c and mean blood glucose show stronger associations with CVD risk factors than do postprandial glycaemia or glucose variability in people with diabetes (the ADAG study). Also, specifically targeting postprandial glucose by using prandial insulin or meglitinides has not unequivocally resulted in improvement of the CVD risk profile. Thus, it is difficult to differentiate between the different indicators of glycaemic control, HbA1c, fasting glucose, postprandial glucose and glucose variability, because they are interlinked, which indicates that the goal should be to achieve glycaemic status as near to normal in all three measures of glycaemic control – fasting glucose, HbA1c and postprandial glucose.

Among other factors, excessive protein glycation and generation of oxidative stress and the induction of inflammation may be underlying mechanisms linking chronic hyperglycaemia and CVD. It has been hypothesized that glycaemic variability may be even more important in the induction of oxidative stress. However, the relationship between glucose variability found in cell cultures and animal studies could not be consistently reproduced in human studies. Differences in methodology used for oxidative stress quantification, as well as differences in duration and frequency of periods with alternating glycaemia, may be possible explanations. The metabolic syndrome may support peripheral inflammation, for instance, by sensitizing leukocytes to up-regulate pro-inflammatory markers in response to glucose, which in turn increases the risk for T2DM and CVDs. These data indicate that effects may vary with type of participant and related metabolic condition. Hence, we need conclusive evidence on the relationship between postprandial glycaemia and CVD from specific intervention studies with cardiovascular outcomes.

### Exercise, postprandial glucose regulation and physical working capacity

Depending on exercise intensity and duration, physical work may have profound effects on the utilization of glucose as a fuel in muscle. During intensive muscle contractions an enhanced glucose uptake from the blood will occur and thus the rate of ‘glucose disappearance’ from the circulation after a meal preceding exercise or taken during exercise will be enhanced. Maintaining high glucose availability can delay fatigue and thereby improve physical as well as mental performance. Several lines of evidence show that intense and lasting muscle work cannot be performed without appropriate availability of carbohydrate and that athletes perform longer at high exercise intensity when glucose availability remains high.

The postprandial effects during exercise, as well as the relative contribution of the individual substrates in the fuel mix used during physical activity, depend on various factors, including the amount of carbohydrate ingested, the timing of intake, and the intensity and duration of exercise. Studies with trained und untrained sedentary individuals found similar metabolic responses following high-glycaemic or low-glycaemic foods and similar differences in substrate metabolism at rest and during low- to moderate-intensity exercise.

The effects of muscle contraction on glucose uptake from the blood are distinct from the effects of insulin and are known to continue even for many hours post-exercise. These effects of exercise lead to both a reduced post-exercise need for insulin production and an enhancement of insulin sensitivity. Because of these observations, regular exercise is recommended as an important measure to manage postprandial blood glucose within ‘more healthy levels’ in patients suffering from T2DM.

### Cognitive function

Studies that report the course of glycaemia following breakfast have, in the main, shown a more favourable outcome for LGI, with one exception. Although young children might be expected to be more susceptible to effects of brief fasting, such as that occurring overnight, because of the greater brain metabolic demands relative to their liver and muscle glycogen stores, and lower capacity for gluconeogenesis, studies are required to confirm the effects of brief (overnight) fasts on cognitive function and the impact of carbohydrate ingestion.

Individuals with impared glucose tolerance or T2DM are also likely to be particularly vulnerable to impairments or less optimal cognitive function because of their elevated metabolic risk factors such as IR, oxidative stress and an impaired glycaemic response, which may all impact negatively on cognitive function. Data on the relationship between postprandial glycaemia and cognitive performance are inconclusive because of methodological limitations of the available studies which suffer from poor description of the meals or products consumed and the cognitive tests performed. There is insufficient information on the standardization of available carbohydrate or the nutrient composition of meals, postprandial glycaemia and the duration of the post-ingestion test period, and studies that explore postprandial glucose in individuals with varying degrees of CVD risk in terms of cognitive impact are required.

In conclusion, there is mechanistic evidence linking postprandial glycaemia and glycaemic variability to maintainance of optimal glycaemic control, the prevention of obesity, diabetes and CVD, to optimizing exercise and cognitive performance. However, data on the importance of postprandial glycaemia *per se* remain, in general, inconclusive because of storng interrelationships with other risk factors, lack of direct measures of glycaemic control and methodological limitations. It is evident that more randomized controlled dietary intervention trials are necessary inducing effective low glucose-response diets to be able to draw more definite conclusions on the role of postprandial glycaemia in relation to health. Such studies should pay close attention to the standardization of diets and include the measurement of postprandial glycaemic profiles over longer periods of time. The relationship between postprandial glucose, diabetes and CVD is a continuum, indicating that early intervention to normalize postprandial glucose (and related indicators of hyperglycaemia and hyperlipidaemia) may be required.

## Conflict of Interest Statement

Ellen E. Blaak, Clare L. Lawton, Jens J. Holst, Signe S. Torekov, Martine Laville, Angela A. Rivellese, Fred Brouns, Inger Björck and Louise Dye received an honorarium from ILSI Europe for their participation in this publication and/or reimbursement of their travel and accommodation costs for attending the related meetings. Agnès Méheust is employed by the European branch of the International Life Sciences Institute (ILSI Europe).

## Footnotes

1 The main authors of this section are Clare Lawton and Jens Juul Holst plus Signe Sørensen Torekov for the incretin parts.

2 The main authors of this section are Ellen Blaak and Michaela Diamant.

3 The main authors of this section are Martine Laville, Sophie Vinoy, Sylvie Normand and Jens Juul Holst, Signe Sørensen Torekov for the incretin parts.

4 The main authors of this section are Angella Rivellese and Lutgarda Bozzetto.

5 The main authors of this section are Fred Brouns and Stephan Theis.

6 The main authors of this section are Inger Bjork, Louise Dye, Dan Lamport and Anne Nilson.

## Abbreviations

ACC: acetyl-CoA carboxylase
ATP: adenosine triphosphate
AUC: area under the curve
BBB: blood–brain barrier
BMI: body mass index
CCK: cholecystokinin
CHD: coronary heart disease
CRP: C-reactive protein
CVD: cardiovascular disease
EI: energy intake
FFA: free fatty acid
GI: glycaemic index
GIP: glucose-dependant insulinotropic polypeptide
GL: glycaemic load
GLP-1: glucagon-like peptide-1
HDL: high-density lipoprotein
HGI: high glycaemic index
HOMA: homeostatic model assessment
IGT: impaired glucose tolerance
IL: interleukin
IMT: intima-media thickness
IPD: individual participant data
LDL: low-density lipoprotein
LGI: low glycaemic index
MAGE: mean amplitude of glycaemic excursions
NO: nitric oxide
OGTT: oral glucose tolerance test
PKC: protein kinase C
PYY: peptide YY
RAGE: advanced glycated end-product receptor
RCT: randomized controlled trial
RR: relative risk
SCFA: short-chain fatty acid
SD: standard deviation
SL: study level
TG: trigylceride
TNFalpha: tumour necrosis factor alpha
